# Anthropometric measures and serum estrogen metabolism in postmenopausal women: the Women’s Health Initiative Observational Study

**DOI:** 10.1186/s13058-017-0810-0

**Published:** 2017-03-11

**Authors:** Hannah Oh, Sally B. Coburn, Charles E. Matthews, Roni T. Falk, Erin S. LeBlanc, Jean Wactawski-Wende, Joshua Sampson, Ruth M. Pfeiffer, Louise A. Brinton, Nicolas Wentzensen, Garnet L. Anderson, JoAnn E. Manson, Chu Chen, Oleg Zaslavsky, Xia Xu, Britton Trabert

**Affiliations:** 1 Division of Cancer Epidemiology and Genetics, National Cancer Institute, 9609 Medical Center Drive, 6E332, Bethesda, MD 20892 USA; 2 Center for Health Research, Kaiser Permanente NW, Portland, OR USA; 3 Department of Epidemiology and Environmental Health, University at Buffalo, Buffalo, NY USA; 4 Program in Epidemiology, Division of Public Health Sciences, Fred Hutchinson Cancer Research Center, Seattle, WA USA; 5 Department of Medicine, Brigham and Women’s Hospital, Harvard Medical School, Boston, MA USA; 6 School of Nursing, University of Washington, Seattle, WA USA; 7 Cancer Research Technology Program, Leidos Biomedical Research, Inc., Frederick National Laboratory for Cancer Research, Frederick, MD USA

**Keywords:** BMI, WHR, Height, Estrogen, Estrogen metabolites, Sex hormones, Postmenopausal

## Abstract

**Background:**

Several anthropometric measures have been associated with hormone-related cancers. However, it is unknown whether estrogen metabolism plays an important role in these relationships. We examined whether measured current body mass index (BMI), waist-to-hip ratio (WHR), height, and self-reported BMI at age 18 years were associated with serum estrogens/estrogen metabolites using baseline, cross-sectional data from 1835 postmenopausal women enrolled in the Women’s Health Initiative Observational Study.

**Methods:**

Fifteen estrogens/estrogen metabolites were quantified using liquid chromatography-tandem mass spectrometry. Geometric means (GMs) of estrogens/estrogen metabolites (in picomoles per liter) were estimated using inverse probability weighted linear regression, adjusting for potential confounders and stratified on menopausal hormone therapy (MHT) use.

**Results:**

Among never or former MHT users, current BMI (≥30 vs. <25 kg/m^2^) was positively associated with parent estrogens (multivariable adjusted GM 432 vs. 239 pmol/L for estrone, 74 vs. 46 pmol/L for estradiol; *p*-trend < 0.001 for both) and all of the 2-, 4-, and 16-pathway estrogen metabolites evaluated (all *p*-trend ≤ 0.02). After additional adjustment for estradiol, unconjugated methylated 2-catechols were inversely associated (e.g., 2-methoxyestrone multivariable GM 9.3 vs. 12.0 pmol/L; *p*-trend < 0.001). Among current MHT users, current BMI was not associated with parent estrogens but was inversely associated with methylated catechols (e.g., 2-methoxyestrone multivariable GM 216 vs. 280 pmol/L; *p*-trend = 0.008). Similar patterns of association were found with WHR; however, the associations were not independent of BMI. Height and BMI at age 18 years were not associated with postmenopausal estrogens/estrogen metabolite levels.

**Conclusions:**

Our data suggest that postmenopausal BMI is associated with increased circulating levels of parent estrogens and reduced methylation of catechol estrogen metabolites, the estrogen metabolism patterns that have previously been associated with higher breast cancer risk.

**Electronic supplementary material:**

The online version of this article (doi:10.1186/s13058-017-0810-0) contains supplementary material, which is available to authorized users.

## Background

Several anthropometric measures have been associated with increased risk of various hormone-related cancers (endometrial, ovarian, and postmenopausal breast cancers) [1–7]. Authors of meta-analyses have estimated a 54% increased risk of endometrial cancer [1] and a 12% increased risk of postmenopausal breast cancer [6] associated with every 5 kg/m^2^ increase in body mass index (BMI). Abdominal adiposity estimated by waist-to-hip ratio (WHR) has also shown strong positive associations with endometrial and postmenopausal breast cancer risk [1, 4], although it is unclear whether the associations are independent of BMI. Further, every 10-cm increase in height has been associated with a 15% higher risk of endometrial [1] and ovarian cancers [3]. One of the hypothesized mechanisms for the BMI associations is that overweight and obese postmenopausal women have elevated levels of circulating estrogens [8], because adipocytes produce estrogens via aromatase activity [9]. Height may indicate early-life nutritional status and exposure to high levels of endogenous proliferative hormones such as estrogens in preadolescence [10].

Estrogens play a key role in endometrial, ovarian, and breast carcinogenesis [11, 12]. Parent estrogens (estradiol and estrone) stimulate cell proliferation largely via estrogen receptor-mediated mechanisms [13]. Parent estrogens are metabolized via the 2-, 4-, and 16-hydroxylation pathways, leading to an array of metabolites in each pathway. Experimental studies have shown that these metabolites can also stimulate cell proliferation via estrogen receptor-mediated mechanisms, and catechols of 2- and 4-pathways, if not methylated, can induce DNA damage directly by forming quinone DNA adducts or indirectly via redox cycling [14–16]. In recent epidemiologic studies, higher levels of parent estrogens and most estrogen metabolites have consistently been associated with higher risk of endometrial and postmenopausal breast cancers [17–20].

Parent estrogens and estrogen metabolites may play an important role in the associations between anthropometric measures and cancer risk. However, beyond parent estrogens [8, 21–23], little is known about the associations between anthropometric measures and circulating estrogen metabolites in postmenopausal women. Further, although current menopausal hormone therapy (MHT) users have higher circulating estrogen/estrogen metabolite levels [24, 25], it is unknown whether anthropometrics are associated with further differences in circulating levels. Using cross-sectional data from the Women’s Health Initiative (WHI) Observational Study (OS), we examined whether current BMI, WHR, height, and BMI at age 18 years were associated with circulating levels of 15 estrogens/estrogen metabolites in postmenopausal women by MHT use.

## Methods

### Study population

This study includes participants in a case-control study of ovarian and endometrial cancers nested within the WHI-OS [20, 26]. The WHI-OS is a prospective cohort study in which researchers recruited 93,676 postmenopausal women aged 50–79 years at 40 clinical centers in the United States between 1993 and 1998 [27, 28]. At the baseline clinic visit, anthropometric measures (height, weight, and waist and hip circumferences) were measured and blood samples were collected. Additionally, baseline self-administered questionnaires were used to collect information on participants’ demographics, medical history, and health behaviors.

Details of the nested case-control study are described elsewhere [20, 26]. In brief, cases were women with ovarian or endometrial cancer diagnosed between study enrollment and 2012. Control subjects were selected from among those women who remained cancer-free at the date of case diagnosis and were frequency-matched to cases on the basis of age at baseline (5-year categories), year of blood draw (1993–1996, 1997–1998), race/ethnicity (white, black, Hispanic, other/unknown), hysterectomy at baseline or during follow-up prior to the index date (for ovarian control subjects only), and MHT use (never, ≤1 year since last MHT use, >1 year since last MHT use, current). Participants had no history of cancer (except nonmelanoma skin cancer), bilateral oophorectomy, or hysterectomy (for endometrial control subjects only), and had ≥1.1 ml of serum available.

A total of 1835 women (507 cases and 450 control subjects among never/former MHT users, 457 cases and 421 control subjects among current MHT users), representing 56,109 women when weighted back to the entire cohort, were included in the study. Because all serum samples were collected at baseline prior to cancer diagnosis (mean 6.7 years from baseline to cancer diagnosis), we included both cases and control subjects in this cross-sectional analysis.

### Exposure assessment

Baseline measured height and weight as well as waist and hip circumferences were used to calculate current BMI (in kilograms per meter squared) and WHR, respectively. BMI at age 18 years was estimated using height and weight at age 18 years recalled via baseline questionnaire. Current BMI was categorized into three groups using the World Health Organization classification cutpoints for overweight and obesity: <25.0 kg/m^2^ (normal), 25.0–29.9 kg/m^2^ (overweight), and ≥30 kg/m^2^ (obese) [29]. In secondary analyses, we subdivided the obesity category by class (class 1: 30.0–34.9 kg/m^2^, class 2: 35.0–39.9 kg/m^2^, class 3: ≥40 kg/m^2^) [29] to further examine the potential dose-response relationship between current BMI and estrogens/estrogen metabolites. WHR, adult height, and BMI at age 18 years were categorized into tertile groups based on the distributions in the entire study population as follows: WHR <0.76, 0.76–0.82, ≥0.83; height <160 cm, 160–164.9 cm, ≥165 cm; and BMI at age 18 years <20 kg/m^2^, 20–21.9 kg/m^2^, ≥22 kg/m^2^.

### Laboratory assays

Aliquoted and batched serum samples were transferred to the Laboratory of Proteomics and Analytical Technologies, Cancer Research Technology Program, Leidos Biomedical Research, Inc. (Frederick, MD, USA), for testing. The assay quantifies the combined concentrations of conjugated and unconjugated forms of 2 parent estrogens (estrone, estradiol) and 13 estrogen metabolites (2-hydroxyestrone, 2-hydroxyestradiol, 2-methoxyestrone, 2-methoxyestradiol, 2-hydroxyestrone-3-methyl ether, 4-hydroxyestrone, 4-methoxyestrone, 4-methoxyestradiol, 16α-hydroxyestrone, estriol, 16-ketoestradiol, 16-epiestriol, 17-epiestriol), as well as the unconjugated concentration of 5 analytes (estrone, estradiol, estriol, 2-methoxyestrone, 2-methoxyestradiol), in serum using a stable isotope dilution liquid chromatography-tandem mass spectrometry (LC-MS/MS) assay [25]. For the five estrogens/estrogen metabolites with unconjugated measurements, their conjugated concentration was calculated by subtracting the unconjugated concentration from the measured (combined) concentration. For the other ten estrogen metabolites, unconjugated concentration was not separately measured, owing to their low abundance in unconjugated forms. (When we do not specify unconjugated or conjugated form, we refer to the combined measured concentration throughout the paper.) Assay reliability was monitored using masked technical replicates across batches. Coefficients of variation were <6% for all analytes; intraclass correlation coefficients (ICCs) ranged from 0.93 to 0.996 (median 0.98) [20, 26]. Correlations among the estrogens/estrogen metabolites ranged from 0.34 to 0.99 [20].

### Statistical analyses

Because estrogen/estrogen metabolite levels vary by MHT use [24, 25], all analyses were stratified on MHT use (*n* = 957 never/former users, *n* = 878 current users). Inverse probability sampling weights were used to adjust the data to represent the population in the entire cohort using methods for secondary phenotype analysis of case-control data described by Li and Gail [30]. Sampling weights were calculated by taking the inverse of sampling fractions: 1 for all cases, and varying weights for control subjects, depending on their strata as defined by matching factors. After log transformation of data to improve normality, geometric means (GMs) in picomoles per liter of individual serum estrogen/estrogen metabolite concentration by exposure categories were estimated using inverse probability weighted linear regression, adjusting for potential confounders: age at blood draw, blood draw year, race, smoking status (never, former, current), and time since menopause (<10 years, 10–19 years, ≥20 years, missing). Additional adjustments for soy and alcohol intake did not change the results and thus were not included in the final models. For current BMI and WHR, we additionally adjusted for physical activity (0, 0.1–9.9, ≥10.0 metabolic equivalents of task-h/week). We performed a test for trend by including the exposure in the model as a continuous variable. The percentage change in GMs between the highest and the lowest categories was estimated by taking the ratio of the GM difference between the two categories over the GM of the lowest category, multiplied by 100. We statistically tested for the difference using a Wald test.

Several secondary analyses were performed. First, for WHR and BMI at age 18 years (Spearman’s *r* ≤ 0.48 with current BMI), we estimated the associations after additional adjustment for current BMI to examine their independent associations. Second, for current BMI among never/former MHT users, we additionally adjusted for unconjugated estradiol to examine whether the associations with individual estrogen metabolites were driven by their correlations with unconjugated estradiol, the estrogen/estrogen metabolite most strongly correlated with current BMI (Spearman’s *r* = 0.46). Next, using pathway groups, we investigated whether current BMI was associated with altered patterns of estrogen metabolism. We compared the mean proportions of parent estrogens out of summed estrogens/estrogen metabolites across BMI categories, with adjustment for the summed concentration of estrogens/estrogen metabolites. Further, because 2-, 4-, and 16-pathway metabolites (“child metabolites”) are metabolized from a limited pool of shared precursors (parent estrogens), an increase in the level of one downstream pathway indicates a reduction in levels of other competing pathways. To address this, we modeled proportions of each child metabolite pathway group (2-catechols [2-hydroxyestrone, 2-hydroxyestradiol]; methylated 2-catechols [2-methoxyestrone and 2-methoxyestradiol]; 4-catechols [4-hydroxyestrone]; methylated 4-catechols [4-methoxyestrone, 4-methoxyestradiol]; 16-pathway metabolites [16α-hydroxyestrone, estriol, 16-ketoestradiol, 16-epiestriol, 17-epiestriol]) out of summed child metabolites, with adjustment for the summed concentration of child metabolites. This approach estimates the association with replacement of one pathway group for other pathway groups while holding summed child metabolites constant. We tested for any difference across BMI categories using a global *F* test; if there was a significant difference (*p* < 0.05), we performed pairwise *t* tests for three different combinations of BMI comparisons (25–29.9 kg/m^2^ vs. <25 kg/m^2^; ≥30 kg/m^2^ vs. <25 kg/m^2^; ≥30 kg/m^2^ vs. 25–29.9 kg/m^2^).

Finally, because subclinical or underlying diseases may influence the associations, we performed sensitivity analyses after excluding endometrial and ovarian cancer cases (*n* = 507 never/former MHT users, *n* = 457 current MHT users); excluding women with a history of diabetes at baseline (*n* = 57 never/former MHT users, *n* = 28 current MHT users); excluding women with low BMI (<18.5 kg/m^2^; *n* = 14 never/former MHT users, *n* = 14 current MHT users); and, among never/former MHT users, excluding women using any potential hormone-related medications (e.g., antiestrogens, glucocorticoid steroids; *n* = 303). To test the robustness of our results, we also repeated analyses after excluding outliers (≤10 values for never/former MHT users, ≤10 values for current MHT users) identified using the extreme Studentized deviate many-outlier procedure [31] and after further stratifying by never vs. former MHT users (*n* = 645 never MHT users, *n* = 312 former MHT users).

All statistical tests were two-sided with a 5% type I error rate. *Q* values reflecting the false discovery rates (FDRs) were calculated to account for multiple comparisons (25 tests per exposure). Analyses were conducted with SAS version 9 software (SAS Institute, Cary, NC, USA).

## Results

### Study population characteristics

The mean age was 64.6 years for never/former MHT users and 61.3 years for current MHT users (Table 1). Most women were white (89% among never/former users, 94% among current users). Compared with never/former MHT users, current users were more likely to have been postmenopausal for <10 years (44% vs. 32%). Mean current BMI, BMI at age 18 years, height, and WHR were similar between the two groups. As expected, serum concentrations of all evaluated estrogens/estrogen metabolites were higher in current MHT users than in never/former users (Tables 2 and 3).

**Table 1:** Characteristics of study population in the Women’s Health Initiative Observational Study

Characteristic	Never or former menopausal hormone therapy users (*n* = 957)			Current menopausal hormone therapy users (*n* = 878)		
	*n*	%	Weighted %^a^	*n*	%	Weighted %^a^
Age at baseline blood draw						
<55 years	82	8.6	8.7	101	11.5	16.8
55–59 years	177	18.5	17.3	194	22.1	25.8
60–64 years	230	24.0	21.0	218	24.8	23.9
65–69 years	201	21.0	23.1	178	20.3	18.3
70–74 years	170	17.8	19.3	135	15.4	11.2
75–79 years	97	10.1	10.6	52	5.9	4.0
Race						
White	841	87.9	89.4	825	94.0	93.5
Year at blood draw						
1993–1996	586	61.2	61.4	544	62.0	61.9
1997–1998	371	38.8	38.6	334	38.0	38.1
Smoking status						
Never	488	51.0	50.2	427	48.6	45.9
Former	402	42.0	41.3	416	47.4	48.8
Current	67	7.0	8.5	35	4.0	5.3
Time since menopause						
<10 years	299	32.9	31.5	349	39.7	44.0
10–19 years	359	39.5	38.7	330	37.6	34.8
≥ 20 years	250	27.5	29.9	199	22.7	21.1
Moderate- to vigorous-intensity physical activity						
0 MET-h/week	205	21.4	19.9	145	16.5	18.4
0.1–9.9 MET-h/week	304	31.8	30.9	279	31.8	34.2
≥10.0 MET-h/week	448	46.8	49.3	454	51.7	47.4
Characteristic	*n*	Weighted *n* ^a^	Weighted mean (SD)	*n*	Weighted *n* ^a^	Weighted mean (SD)
Current BMI, kg/m^2^	953	30,814	27.0 (0.27)	877	25,152	26.6 (0.28)
BMI at age 18 years, kg/m^2^	933	30,149	20.6 (0.15)	859	24,661	20.5 (0.12)
Waist-to-hip ratio	952	30,720	0.81 (0.004)	877	25,152	0.79 (0.004)
Height, cm	956	30,817	162.2 (0.30)	877	25,152	163.1 (0.34)

*BMI* Body mass index, *MET* Metabolic equivalent of task

^a^Weighted percentages and weighted n reflect weighted counts and refer to the study cohort

**Table 2:** Geometric means (pmol/L) and 95% CIs of serum estrogens/estrogen metabolites by current body mass index in postmenopausal women not using menopausal hormone therapy in the Women’s Health Initiative Observational Study

	Model 1^a^						Model 1 + unconjugated estradiol^b^					
	Geometric mean (95% CI)			*p*-trend^c^	%Δ^d^	*p*-diff^e^	Geometric mean (95% CI)			*p*-trend^c^	%Δ^d^	*p*-diff^e^
	<25 kg/m^2^	25–29.9 kg/m^2^	≥30 kg/m^2^				<25 kg/m^2^	25–29.9 kg/m^2^	≥30 kg/m^2^			
Median, kg/m^2^	22.5	27.1	33.6				22.5	27.1	33.6			
*n*	345	294	314				345	294	314			
Weighted *n* ^f^	13,571	9351	7893				13,571	9351	7893			
Estrone	239 (201–284)	337 (281–404)	432 (355–525)	*<0.001* ^g^	80.8	*<0.001* ^g^	313 (266–368)	344 (296–399)	326 (280–380)	0.91	4.2	0.64
Conjugated	179 (147–218)	265 (217–324)	348 (281–431)	*<0.001* ^g^	94.4	*<0.001* ^g^	237 (197–284)	271 (229–320)	260 (218–310)	0.59	9.7	0.35
Unconjugated	51.3 (45.0–58.6)	62.4 (55.1–70.6)	75.6 (65.2–87.8)	*<0.001* ^g^	47.4	*<0.001* ^g^	64.4 (57.5–72.1)	63.4 (58.0–69.3)	59.8 (54.2–65.9)	0.13	−7.1	0.24
Estradiol	45.9 (37.6–56.1)	59.0 (48.3–72.2)	73.9 (60.0–91.0)	*<0.001* ^g^	61.0	*<0.001* ^g^	60.7 (52.5–70.2)	60.3 (52.7–68.8)	55.3 (48.7–62.7)	0.33	−8.9	0.19
Conjugated	31.0 (25.2–38.2)	40.1 (32.3–49.8)	45.9 (36.6–57.6)	*<0.001* ^g^	48.1	*<0.001* ^g^	38.5 (31.7–46.9)	40.7 (34.1–48.7)	36.6 (30.6–43.8)	0.99	−4.9	0.61
Unconjugated	10.2 (8.13–12.7)	14.9 (12.2–18.2)	23.5 (18.8–29.4)	*<0.001* ^g^	130	*<0.001* ^g^	NA	NA	NA	NA	NA	NA
2-Hydroxyestrone	55.6 (47.8–64.8)	71.7 (61.5–83.5)	82.9 (70.5–97.4)	*<0.001* ^g^	49.1	*<0.001* ^g^	66.1 (56.7–77.0)	72.6 (63.3–83.3)	69.3 (59.6–80.6)	0.83	4.8	0.59
2-Hydroxyestradiol	14.2 (12.3–16.5)	17.8 (15.2–20.7)	20.4 (17.3–24.0)	*<0.001* ^g^	43.7	*<0.001* ^g^	16.8 (14.4–19.5)	18.0 (15.6–20.6)	17.2 (14.8–20.1)	0.69	2.4	0.76
2-Methoxyestrone	37.8 (33.2–43.0)	43.6 (38.4–49.5)	47.7 (41.6–54.6)	*0.001* ^g^	26.2	*0.001* ^g^	44.6 (39.5–50.3)	44.2 (39.4–49.5)	40.2 (35.6–45.4)	0.05	−9.9	0.15
Conjugated	27.5 (23.9–31.6)	32.2 (27.9–37.1)	34.5 (29.7–40.2)	*0.002* ^g^	25.5	*0.004* ^g^	31.8 (27.9–36.2)	32.5 (28.6–37.0)	29.7 (25.8–34.2)	0.25	−6.6	0.42
Unconjugated	9.70 (8.39–11.2)	10.5 (9.13–12.0)	11.6 (10.0–13.5)	*0.02* ^h^	19.6	*0.03* ^h^	12.0 (10.5–13.8)	10.6 (9.29–12.2)	9.29 (8.09–10.7)	*<0.001* ^g^	−22.6	*0.001* ^h^
2-Methoxyestradiol	11.4 (9.63–13.5)	13.5 (11.5–15.8)	16.4 (13.7–19.7)	*<0.001* ^g^	43.9	*<0.001* ^g^	13.7 (11.8–16.0)	13.7 (12.0–15.6)	13.6 (11.5–16.0)	0.50	−0.7	0.91
Conjugated	9.02 (7.49–10.9)	10.7 (8.88–12.9)	13.4 (11.0–16.4)	*<0.001* ^g^	48.6	*<0.001* ^g^	10.8 (9.01–12.8)	10.9 (9.17–12.8)	11.2 (9.27–13.5)	0.83	3.7	0.70
Unconjugated	1.91 (1.63–2.23)	2.21 (1.90–2.57)	2.36 (2.02–2.76)	*<0.001* ^g^	23.6	*0.006* ^g^	2.37 (2.12–2.65)	2.24 (1.99–2.53)	1.88 (1.69–2.09)	*0.002* ^h^	−20.7	*<0.001* ^g^
2-Hydroxyestrone-3-methyl ether	6.74 (5.93–7.67)	7.94 (6.89–9.14)	8.72 (7.59–10.0)	*0.004* ^g^	29.4	*<0.001* ^g^	7.68 (6.71–8.79)	8.01 (7.02–9.15)	7.62 (6.62–8.77)	0.24	−0.8	0.92
4-Hydroxyestrone	6.77 (5.80–7.89)	8.69 (7.47–10.1)	10.2 (8.57–12.1)	*<0.001* ^g^	50.7	*<0.001* ^g^	7.97 (6.82–9.32)	8.80 (7.65–10.1)	8.57 (7.27–10.1)	0.96	7.5	0.44
4-Methoxyestrone	3.93 (3.45–4.48)	4.36 (3.87–4.92)	4.86 (4.18–5.65)	*0.02* ^h^	23.7	*0.008* ^g^	4.47 (3.89–5.13)	4.40 (3.91–4.96)	4.25 (3.65–4.94)	0.19	−4.9	0.58
4-Methoxyestradiol	1.66 (1.41–1.95)	1.87 (1.62–2.16)	2.22 (1.86–2.65)	*<0.001* ^g^	33.7	*<0.001* ^g^	1.95 (1.66–2.29)	1.89 (1.65–2.16)	1.87 (1.57–2.22)	0.30	−4.1	0.64
16α-Hydroxyestrone	27.5 (23.4–32.4)	35.8 (30.6–42.0)	41.6 (35.0–49.4)	*<0.001* ^g^	51.3	*<0.001* ^g^	32.8 (27.8–38.6)	36.3 (31.4–41.9)	34.7 (29.5–40.8)	0.92	5.8	0.54
Estriol	114 (97.0–133)	155 (133–181)	178 (151–211)	*<0.001* ^g^	56.1	*<0.001* ^g^	137 (117–160)	157 (137–181)	147 (125–172)	0.94	7.3	0.45
Conjugated	87.1 (73.0–104)	124 (105–147)	143 (118–172)	*<0.001* ^g^	64.2	*<0.001* ^g^	105 (88.2–125)	126 (108–147)	118 (98.3–141)	0.60	12.4	0.28
Unconjugated	23.2 (20.1–26.6)	27.4 (24.0–31.4)	33.0 (28.3–38.4)	*<0.001* ^g^	42.2	*<0.001* ^g^	27.9 (24.5–31.7)	27.8 (24.5–31.6)	27.2 (23.6–31.3)	0.29	−2.5	0.74
16-Ketoestradiol	29.1 (24.7–34.3)	38.2 (32.4–45.1)	44.3 (37.2–52.9)	*<0.001* ^g^	52.2	*<0.001* ^g^	34.7 (29.4–40.9)	38.7 (33.3–45.0)	37.0 (31.3–43.7)	0.95	6.6	0.50
16-Epiestriol	12.9 (11.1–14.9)	16.4 (14.0–19.2)	19.3 (16.5–22.6)	*<0.001* ^g^	49.6	*<0.001* ^g^	15.1 (13.1–17.4)	16.6 (14.4–19.1)	16.4 (14.1–19.0)	0.78	8.6	0.31
17-Epiestriol	10.8 (9.37–12.3)	13.5 (11.6–15.6)	16.4 (14.0–19.3)	*<0.001* ^g^	51.9	*<0.001* ^g^	12.6 (11.0–14.4)	13.6 (12.0–15.5)	13.9 (12.0–16.2)	0.72	10.3	0.21

*NA* Not applicable

^a^Model 1: adjusted for age at blood draw (<55, 55–59, 60–64, 65–69, 70–74, 75–79 years), blood draw year (1993–1996, 1997–1998), race (white, nonwhite), smoking status (never, former, current), time since menopause (<10 years, 10–19 years, ≥20 years, missing), moderate- to vigorous-intensity physical activity (0, 0.1–9.9, ≥10 metabolic equivalents of task-h/week)

^b^Model 1 + unconjugated estradiol (log-transformed, continuous)

^c^ *p*-trend was estimated using the Wald test for continuous body mass index (BMI; in kilograms per meter squared)

^d^%Δ indicates the percentage change in estrogen/estrogen metabolite levels, comparing women with current BMI ≥30 vs. <25 kg/m^2^, and was estimated by taking the ratio of the geometric mean difference in estrogen/estrogen metabolite levels between women with current BMI ≥30 vs. <25 kg/m^2^ to the geometric mean of women with current BMI <25 kg/m^2^, multiplied by 100

^e^ *p*-diff was estimated using the Wald test and indicates a *p*-value for comparing estrogen/estrogen metabolite levels of women with current BMI ≥30 vs. <25 kg/m^2^

^f^Weighted *n* reflects weighted counts and refers to the study cohort

^g^False discovery rate (FDR) *q* value <0.01

^h^FDR *q* values <0.05 and ≥0.01

Note: Italized *p*-values indicate nominal statistical significance at 0.05

**Table 3:** Geometric means (pmol/L) and 95% CIs of serum estrogens/estrogen metabolites by current body mass index in postmenopausal women currently using menopausal hormone therapy in the Women’s Health Initiative Observational Study

	Geometric means (95% CI)^a^			*p*-trend^b^	%Δ^c^	*p*-diff^d^
	<25 kg/m^2^	25–29.9 kg/m^2^	≥30 kg/m^2^			
Median (kg/m^2^)	22.4	27.2	33.1			
*n*	445	257	175			
Weighted *n* ^e^	11,498	7844	5810			
Estrone	3201 (2433–4212)	2668 (1960–3632)	3201 (2291–4472)	0.46	0.0	1.00
Conjugated	2930 (2208–3887)	2473 (1801–3396)	2896 (2046–4098)	0.48	−1.2	0.94
Unconjugated	227 (186–277)	191 (150–244)	205 (158–264)	0.18	−9.7	0.38
Estradiol	444 (339–582)	390 (292–522)	414 (300–572)	0.13	−6.8	0.62
Conjugated	368 (273–497)	327 (237–450)	341 (238–486)	0.13	−7.3	0.63
Unconjugated	42.5 (33.8–53.5)	42.9 (33.1–55.7)	50.8 (38.5–66.9)	0.15	19.5	0.17
2-Hydroxyestrone	462 (378–565)	382 (300–486)	422 (331–539)	0.21	−8.7	0.42
2-Hydroxyestradiol	109 (90.6–131)	89.1 (70.9–112)	98.3 (77.7–124)	0.16	−9.8	0.35
2-Methoxyestrone	280 (236–333)	219 (180–266)	216 (174–268)	*0.008*	−22.9	*0.02*
Conjugated	174 (142–215)	135 (108–169)	139 (109–177)	*0.04*	−20.1	0.05
Unconjugated	78.8 (62.4–99.5)	62.5 (45.6–85.8)	56.3 (41.3–76.8)	*<0.001* ^f^	−28.6	*0.02*
2-Methoxyestradiol	92.6 (72.7–118)	70.1 (54.1–90.8)	73.7 (57.4–94.6)	*0.02*	−20.4	*0.03*
Conjugated	80.9 (62.0–105)	58.3 (43.8–77.5)	60.8 (46.3–79.7)	*0.001* ^g^	−24.8	*0.01*
Unconjugated	8.90 (7.56–10.5)	8.43 (6.86–10.4)	8.08 (6.49–10.1)	0.19	−9.2	0.38
2-Hydroxyestrone-3-methyl ether	44.2 (36.7–53.2)	34.8 (28.3–42.6)	36.8 (29.3–46.3)	*0.04*	−16.7	0.09
4-Hydroxyestrone	61.6 (50.2–75.5)	51.3 (40.2–65.4)	57.0 (44.4–73.1)	0.26	−7.5	0.50
4-Methoxyestrone	28.9 (24.2–34.6)	23.1 (19.0–28.0)	23.1 (18.7–28.6)	*0.02*	−20.1	*0.04*
4-Methoxyestradiol	12.3 (9.51–16.0)	9.61 (7.26–12.7)	9.98 (7.52–13.2)	*0.04*	−18.9	0.07
16α-Hydroxyestrone	247 (200–304)	200 (155–257)	227 (177–292)	0.28	−8.1	0.48
Estriol	1136 (897–1438)	966 (740–1261)	1075 (812–1424)	0.36	−5.4	0.67
Conjugated	988 (772–1264)	844 (638–1117)	914 (680–1227)	0.35	−7.5	0.57
Unconjugated	129 (106–158)	113 (89.3–142)	116 (90.3–148)	0.12	−10.1	0.29
16-Ketoestradiol	286 (231–354)	229 (178–295)	262 (203–339)	0.30	−8.4	0.47
16-Epiestriol	86.7 (71.1–106)	70.2 (56.5–87.3)	79.5 (62.7–101)	0.21	−8.3	0.44
17-Epiestriol	58.2 (47.6–71.3)	46.5 (37.2–58.1)	51.9 (40.6–66.4)	0.28	−10.8	0.31

^a^Adjusted for age at blood draw (<55, 55–59, 60–64, 65–69, 70–74, 75–79 years), blood draw year (1993–1996, 1997–1998), race (white, nonwhite), smoking status (never, former, current), time since menopause (<10 years, 10–19 years, ≥20 years, missing), moderate- to vigorous-intensity physical activity (0, 0.1–9.9, ≥10 metabolic equivalents of task-h/week)

^b^ *p*-trend was estimated using the Wald test for continuous body mass index (BMI; kilograms per meter squared)

^c^%Δ indicates the percentage change in estrogen/estrogen metabolite levels, comparing women with current BMI ≥30 vs. <25 kg/m^2^, and was estimated by taking the ratio of the geometric mean difference in estrogen/estrogen metabolite levels between women with current BMI ≥30 vs. <25 kg/m^2^ to the geometric mean of women with current BMI <25 kg/m^2^, multiplied by 100

^d^ *p*-diff was estimated using the Wald test and indicates a *p* value for comparing estrogen/estrogen metabolite levels of women with current BMI ≥30 vs. <25 kg/m^2^

^e^Weighted *n* reflects weighted counts and refers to the study cohort

^f^False discovery rate (FDR) *q* value <0.01

^g^FDR *q* values <0.05 and ≥0.01

Note: Italized *p*-values indicate nominal statistical significance at 0.05

### Current body mass index

Among never/former MHT users, higher current BMI (≥30 vs. <25 kg/m^2^) was significantly associated with higher levels of parent estrogens (GM 432 vs. 239 pmol/L for estrone, 73.9 vs. 45.9 pmol/L for estradiol; *p*-trend < 0.001) and all of the 2-, 4-, and 16-pathway metabolites evaluated (all *p*-trend ≤ 0.02) (Table 2). However, after adjustment for unconjugated estradiol, these patterns did not persist, and inverse associations were observed for unconjugated methylated 2-catechols (9.29 vs. 12.0 pmol/L, *p*-trend < 0.001 for unconjugated 2-methoxyestrone; 1.88 vs. 2.37 pmol/L, *p*-trend = 0.002 for unconjugated 2-methoxyestradiol) (Table 2). Similar dose-response trends were found across classes 1–3 obesity (Additional file 1: Table S1).

Among current MHT users, current BMI (≥30 vs. <25 kg/m^2^) was not associated with parent estrogens (3201 vs. 3201 pmol/L for estrone; 414 vs. 444 pmol/L for estradiol; *p*-trend ≥ 0.13), but it was inversely associated with methylated catechols (216 vs. 280 pmol/L, *p*-trend = 0.008 for 2-methoxyestrone; 73.7 vs. 92.6 pmol/L, *p*-trend = 0.02 for 2-methoxyestradiol; 23.1 vs. 28.9 pmol/L, *p*-trend = 0.02 for 4-methoxyestrone) (Table 3).

**Fig. 1:**
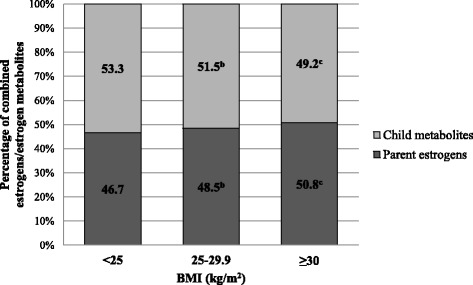
Percentages of parent estrogens (estradiol and estrone) and child estrogen metabolites (2-, 4-, and 16-hydroxylation pathway metabolites) out of summed estrogens/estrogen metabolites by current body mass index (BMI) among never or former menopausal hormone therapy users^a^. Adjusted for age at blood draw (<55, 55-59, 60-64, 65-69, 70-74, 75-79 years), blood draw year (1993-1996, 1997-1998), race (white, non-white), smoking status (never, former, current), time since menopause (<10, 10-19, ≥20 years, missing), moderate- to vigorous-intensity physical activity (0, 0.1-9.9, ≥10 MET-hr/wk), summed concentration of estrogens/estrogen metabolites (continuous, pmol/L).Note: Summed estrogens/estrogen metabolites include the summed concentration of parent estrogens (estrone, estradiol) and child metabolites (2-hydroxyestrone, 2-hydroxyestradiol, 2-methoxyestrone, 2-methoxyestradiol, 2-hyroxyestrone-3-methyl ether, 4-hydroxyestrone, 4-methoxyestrone, 4-methoxyestradiol, 16α-hydroxyestrone, estriol, 16-ketoestradiol, 16-epiestriol, 17-epiestriol).^a^Overall F-test *p*=0.02.^b^ *P*-value for comparing percent parent estrogens between women with current BMI 25-29.9 vs. <25 kg/m^2^ was 0.11.^c^ *P*-value for comparing percent parent estrogens between women with current BMI ≥30 vs. 25-29.9 kg/m^2^ was 0.10. *P*-value for comparing percent parent estrogens between women with current BMI ≥30 vs. <25 kg/m^2^ was 0.006.*BMI* body mass index

**Fig. 2:**
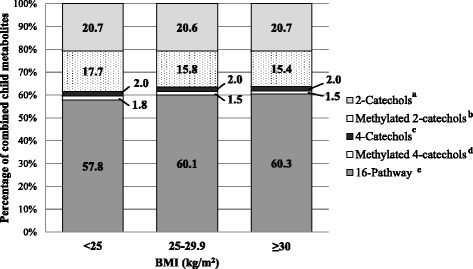
Percentages of each pathway estrogen metabolites (2-catechols, methylated 2-catechols, 4-catechols, methylated 4-catechols, 16-pathway metabolites) out of summed child estrogen metabolites by current body mass index (BMI) among never/former menopausal hormone therapy users. Adjusted for age at blood draw (<55, 55-59, 60-64, 65-69, 70-74, 75-79 years), blood draw year (1993-1996, 1997-1998), race (white, non-white), smoking status (never, former, current), time since menopause (<10, 10-19, ≥20 years, missing), moderate- to vigorous-intensity physical activity (0, 0.1-9.9, ≥10 MET-hr/wk), summed concentration of child estrogen metabolites (continuous, pmol/L). Note: Summed child estrogen metabolites include the following estrogen metabolites: 2-catechols (2-hydroxyestrone, 2-hydroxyestradiol), methylated 2-catechols (conjugated and unconjugated 2-methoxyestrone, conjugated and unconjugated 2-methoxyestradiol, 2-hyroxyestrone-3-methyl ether), 4-catechols (4-hydroxyestrone), methylated 4-catechols (4-methoxyestrone, 4-methoxyestradiol), 16-pathway metabolites (16α-hydroxyestrone, conjugated and unconjugated estriol, 16-ketoestradiol, 16-epiestriol, 17-epiestriol). ^a^For 2-catechols, overall F-test *p*=0.89. ^b^For methylated 2-catechols, overall F-test p=0.001. P-values for comparing women with current BMI 25-29.9 vs. <25 kg/m^2^ was 0.002, comparing women with current BMI ≥30 vs. 25-29.9 kg/m^2^ was 0.57, and comparing women with current BMI ≥30 vs. <25 kg/m^2^ was 0.001. ^c^For 4-catechols, overall F-test p=0.59. ^d^For methylated 4-catechols, overall F-test p=0.0002. P-values for comparing women with current BMI 25-29.9 vs. <25 kg/m^2^ was <0.0001, comparing women with current BMI ≥30 vs. 25-29.9 kg/m^2^ was 0.82, and comparing women with current BMI ≥30 vs. <25 kg/m^2^ was 0.003. ^e^For 16-pathway metabolites, overall F-test p<0.0001. P-values for comparing women with current BMI 25-29.9 vs. <25 kg/m^2^ was 0.0003, comparing women with current BMI ≥30 vs. 25-29.9 kg/m^2^ was 0.78, and comparing women with current BMI ≥30 vs. <25 kg/m^2^ was 0.0002.*BMI* body mass index

In secondary analyses among never/former MHT users, the proportion of parent estrogens was significantly higher in obese women (BMI ≥30 vs. <25 kg/m^2^, 50.8% vs. 46.7%, respectively; *p* = 0.006) (Fig. 1). Further, obese women had lower proportions of methylated 2-catechols (15.4% vs. 17.7%; *p* = 0.001) and methylated 4-catechols (1.5% vs. 1.8%; *p* = 0.003) and higher proportions of 16-pathway metabolites (60.3% vs. 57.8%; *p* = 0.0002) (Fig. 2). Similar results were found in overweight women; however, there was no statistically significant difference in proportions between overweight and obese women. Results were similar among current MHT users, although the association with proportion of parent estrogens was statistically nonsignificant (BMI ≥30 vs. <25 kg/m^2^, 56.4% vs. 54.3%, respectively; overall *F* test *p* = 0.40) (Additional file 2: Figure S1, Additional file 3: Figure S2).

### Waist-to-hip ratio

Among never/former MHT users, WHR (≥0.83 vs. <0.76) was positively associated with parent estrogens (348 vs. 286 pmol/L for estrone, 62.5 vs. 50.2 pmol/L for estradiol; *p*-trend ≤ 0.01) and nearly all of the 16-pathway metabolites; however, in models mutually adjusting for current BMI and WHR, only current BMI remained significantly associated (Table 4). Among current MHT users, WHR was not associated with parent estrogens but was inversely associated with methylated catechols, namely 2-methoxyestrone (219 vs. 272 pmol/L, *p*-trend = 0.03), 2-hydroxyestrone-3-methyl ether (35.4 vs. 44.1 pmol/L, *p*-trend = 0.03), and 4-methoxyestrone (22.8 vs. 28.6 pmol/L, *p*-trend = 0.04); the associations did not remain significant after adjustment for current BMI (Additional file 4: Table S2).

**Table 4:** Geometric means (pmol/L) and 95% CIs of serum estrogens/estrogen metabolites by waist-to-hip ratio in postmenopausal women not using menopausal hormone therapy in the Women’s Health Initiative Observational Study

	Model 1^a^						Model 1 + current BMI^b^					
	Geometric means (95% CI)			*p*-trend^c^	%Δ^d^	*p*-diff^e^	Geometric means (95% CI)			*p*-trend^c^	%Δ^d^	*p*-diff^e^
	<0.76	0.76–0.82	≥0.83				<0.76	0.76–0.82	≥0.83			
Median	0.74	0.79	0.87				0.74	0.79	0.87			
*n*	229	340	383				229	340	383			
Weighted *n* ^f^	7616	10,905	12,199				7616	10,905	12,199			
Estrone	286 (231–354)	290 (243–347)	348 (292–415)	*0.003* ^g^	21.7	0.07	345 (276–431)	320 (268–382)	327 (276–388)	0.80	−5.2	0.64
Conjugated	216 (170–274)	229 (188–279)	271 (223–329)	*0.002* ^g^	25.5	0.06	265 (207–341)	255 (209–312)	253 (209–306)	0.77	−4.5	0.69
Unconjugated	62.4 (53.6–72.5)	53.0 (46.4–60.6)	66.7 (58.7–75.8)	0.05	6.9	0.39	70.8 (61.1–82.0)	56.7 (49.9–64.4)	63.9 (56.6–72.2)	0.53	−9.7	0.19
Estradiol	50.2 (39.5–63.8)	53.1 (43.9–64.2)	62.5 (51.3–76.1)	*0.01* ^g^	24.5	*0.04*	59.0 (47.0–74.1)	57.8 (48.1–69.5)	59.2 (48.7–72.0)	0.98	0.3	0.98
Conjugated	31.8 (24.7–40.9)	36.5 (30.0–44.6)	40.6 (32.8–50.3)	*0.04*	27.7	*0.04*	36.4 (28.5–46.5)	39.2 (32.3–47.7)	38.8 (31.4–48.1)	0.88	6.6	0.59
Unconjugated	13.9 (10.7–18.2)	11.6 (9.20–14.5)	17.5 (14.3–21.4)	*<0.001* ^h^	25.9	*0.04*	18.5 (14.5–23.4)	13.4 (10.9–16.5)	15.9 (13.2–19.3)	0.89	−14.1	0.19
2-Hydroxyestrone	62.6 (52.1–75.1)	64.5 (55.7–74.7)	72.1 (62.2–83.7)	*0.01* ^g^	15.2	0.11	69.9 (57.9–84.4)	68.4 (58.9–79.3)	69.5 (59.8–80.8)	0.64	−0.6	0.95
2-Hydroxyestradiol	16.0 (13.4–19.1)	16.3 (14.1–18.9)	17.8 (15.3–20.6)	0.05	11.3	0.23	17.8 (14.8–21.3)	17.3 (14.9–20.0)	17.2 (14.8–20.0)	0.96	−3.4	0.71
2-Methoxyestrone	45.4 (39.0–52.7)	40.2 (35.5–45.7)	43.1 (38.1–48.7)	0.69	−5.1	0.51	49.3 (42.1–57.7)	42.0 (37.0–47.8)	41.9 (37.1–47.5)	0.18	−15.0	0.05
Conjugated	32.0 (27.1–37.9)	30.3 (26.4–34.7)	31.1 (27.1–35.6)	0.64	−2.8	0.73	34.9 (29.3–41.5)	31.7 (27.6–36.3)	30.2 (26.4–34.7)	0.26	−13.5	0.12
Unconjugated	12.4 (10.5–14.7)	9.21 (7.96–10.7)	10.9 (9.61–12.3)	1.00	−12.1	0.12	13.3 (11.2–15.8)	9.56 (8.24–11.1)	10.6 (9.38–12.0)	0.22	−20.3	*0.01*
2-Methoxyestradiol	13.3 (11.1–16.0)	12.7 (10.8–14.9)	14.0 (11.8–16.5)	*0.04*	5.3	0.62	15.0 (12.5–18.0)	13.5 (11.5–15.9)	13.4 (11.4–15.9)	0.98	−10.7	0.22
Conjugated	10.4 (8.49–12.6)	10.2 (8.47–12.2)	11.2 (9.32–13.6)	*0.02* ^g^	7.7	0.39	11.7 (9.54–14.3)	10.8 (9.02–13.0)	10.8 (8.94–13.1)	0.70	−7.7	0.44
Unconjugated	2.41 (2.01–2.89)	1.96 (1.69–2.27)	2.17 (1.88–2.50)	0.96	−10.0	0.20	2.69 (2.26–3.20)	2.07 (1.80–2.40)	2.10 (1.81–2.42)	0.06	−21.9	*0.003*
2-Hydroxyestrone-3-methyl ether	7.80 (6.62–9.19)	7.29 (6.40–8.31)	7.89 (6.96–8.95)	0.31	1.2	0.89	8.35 (7.01–9.94)	7.56 (6.60–8.65)	7.72 (6.79–8.77)	0.76	−7.5	0.36
4-Hydroxyestrone	7.66 (6.35–9.23)	7.79 (6.71–9.03)	8.82 (7.59–10.2)	*0.01* ^g^	15.1	0.13	8.57 (7.05–10.4)	8.26 (7.10–9.60)	8.50 (7.30–9.89)	0.48	−0.8	0.93
4-Methoxyestrone	4.60 (3.96–5.35)	4.01 (3.55–4.54)	4.46 (3.92–5.08)	0.56	−3.0	0.71	4.90 (4.19–5.73)	4.15 (3.66–4.71)	4.37 (3.84–4.98)	0.51	−10.8	0.17
4-Methoxyestradiol	1.95 (1.62–2.34)	1.74 (1.49–2.02)	1.95 (1.67–2.29)	0.22	0.0	0.97	2.14 (1.79–2.57)	1.82 (1.57–2.13)	1.89 (1.61–2.22)	0.74	−11.7	0.19
16α-Hydroxyestrone	31.2 (25.7–37.8)	32.0 (27.4–37.3)	36.0 (30.8–42.1)	*0.01* ^g^	15.4	0.13	35.0 (28.7–42.8)	34.0 (29.0–39.7)	34.7 (29.6–40.7)	0.60	−0.9	0.92
Estriol	130 (107–159)	133 (114–155)	156 (134–181)	*0.005* ^g^	20.0	0.07	148 (120–181)	142 (121–166)	149 (129–173)	0.46	0.7	0.90
Conjugated	97.3 (78.1–121)	106 (89.8–126)	123 (105–146)	*0.002* ^g^	26.4	*0.03*	111 (89.0–140)	114 (96.2–136)	118 (99.8–139)	0.34	6.3	0.61
Unconjugated	29.3 (24.8–34.7)	23.3 (20.3–26.8)	29.1 (25.5–33.1)	0.13	−0.7	0.92	32.7 (27.5–39.0)	24.7 (21.5–28.4)	28.0 (24.6–31.9)	0.67	−14.4	0.09
16-Ketoestradiol	33.5 (27.5–40.8)	33.6 (28.7–39.3)	38.5 (32.9–45.1)	*0.01* ^g^	14.9	0.16	37.6 (30.6–46.2)	35.7 (30.4–42.0)	37.1 (31.5–43.6)	0.53	−1.3	0.88
16-Epiestriol	14.4 (12.1–17.1)	14.8 (12.8–17.1)	16.7 (14.4–19.4)	*0.02* ^g^	16.0	0.07	16.1 (13.4–19.3)	15.7 (13.5–18.2)	16.1 (13.9–18.7)	0.93	0.0	0.99
17-Epiestriol	12.1 (10.2–14.4)	12.1 (10.5–13.9)	14.1 (12.2–16.3)	*0.01* ^g^	16.5	0.07	13.6 (11.4–16.2)	12.9 (11.1–14.9)	13.6 (11.8–15.7)	0.84	0.0	0.98

^a^Model 1: adjusted for age at blood draw (<55, 55–59, 60–64, 65–69, 70–74, 75–79 years), blood draw year (1993–1996, 1997–1998), race (white, nonwhite), smoking status (never, former, current), time since menopause (<10 years, 10–19 years, ≥20 years, missing), moderate- to vigorous-intensity physical activity (0, 0.1–9.9, ≥10 metabolic equivalents of task-h/week)

^b^Model 1 + current body mass index (kg/m^2^, continuous)

^c^ *p*-trend was estimated using the Wald test for continuous waist-to-hip ratio

^d^%Δ indicates the percentage change in estrogen/estrogen metabolite levels, comparing women with waist-to-hip ratio ≥0.83 vs. <0.76, and was estimated by taking the ratio of the geometric mean difference in estrogen/estrogen metabolite levels between women with waist-to-hip ratio ≥0.83 vs. <0.76 to the geometric mean of women with waist-to-hip ratio <0.76, multiplied by 100

^e^ *p*-diff was estimated using the Wald test and indicates a *p* value for comparing estrogen/estrogen metabolite levels of women with waist-to-hip ratio ≥0.83 vs. <0.76

^f^Weighted *n* reflects weighted counts and refers to the study cohort

^g^False discovery rate (FDR) *q* values <0.05 and ≥0.01

^h^FDR *q* value <0.01

Note: Italized *p*-values indicate nominal statistical significance at 0.05

### Height

Height (≥165 vs. <160 cm) was not associated with circulating estrogens/estrogen metabolites in postmenopausal women, except for inverse associations with conjugated 2-methoxyestrone (27.0 vs. 33.2 pmol/L, *p*-trend = 0.01) and 4-methoxyestrone (3.90 vs. 4.59 pmol/L, *p*-trend = 0.03) among never/former MHT users (Table 5).

**Table 5:** Geometric means (pmol/L) and 95% CIs of serum estrogens/estrogen metabolites by height in postmenopausal women in the Women’s Health Initiative Observational Study

	Never and former menopausal hormone therapy users						Current menopausal hormone therapy users					
	Geometric means (95% CI)^a^			*p*-trend^b^	%Δ^c^	*p*-diff^d^	Geometric means (95% CI)^a^			*p*-trend^b^	%Δ^c^	*p*-diff^d^
	<160 cm	160–164.9 cm	≥165 cm				<160 cm	160–164.9 cm	≥165 cm			
Median, cm	155.9	162.4	167.9				156.5	162.5	168.7			
*n*	333	310	313				289	269	319			
Weighted *n* ^e^	10,171	10,625	10,021				7764	7588	9800			
Estrone	288 (240–345)	338 (285–402)	300 (241–373)	0.27	4.2	0.67	3256 (2420–4383)	2926 (2079–4117)	3019 (2215–4117)	0.77	−7.3	0.57
Conjugated	220 (180–270)	267 (221–324)	233 (183–297)	0.25	5.9	0.61	2963 (2185–4018)	2735 (1920–3895)	2728 (1985–3750)	0.69	−7.9	0.55
Unconjugated	58.6 (51.1–67.2)	62.6 (55.0–71.4)	57.7 (49.4–67.5)	0.69	−1.5	0.85	229 (181–289)	196 (152–253)	213 (167–271)	0.94	−7.0	0.51
Estradiol	53.3 (42.9–66.1)	61.2 (50.7–73.9)	51.6 (41.4–64.4)	0.51	−3.2	0.75	443 (327–600)	399 (288–555)	425 (313–578)	0.91	−4.1	0.75
Conjugated	34.9 (27.6–44.1)	41.2 (33.9–50.1)	33.5 (26.6–42.1)	0.65	−4.0	0.69	370 (266–515)	335 (233–482)	346 (247–483)	0.64	−6.5	0.63
Unconjugated	13.8 (11.0–17.2)	14.9 (12.0–18.6)	13.8 (10.7–17.8)	0.45	0.0	0.99	45.5 (35.5–58.3)	42.2 (32.2–55.3)	43.0 (33.3–55.7)	0.41	−5.5	0.64
2-Hydroxyestrone	64.9 (55.1–76.5)	69.5 (60.1–80.3)	62.8 (53.1–74.2)	0.98	−3.2	0.68	448 (358–560)	407 (315–527)	439 (347–556)	0.90	−2.0	0.86
2-Hydroxyestradiol	16.2 (13.7–19.1)	17.4 (15.1–20.0)	16.0 (13.5–18.8)	0.88	−1.2	0.86	103 (82.9–128)	96.9 (76.3–123)	105 (83.8–132)	0.82	1.9	0.87
2-Methoxyestrone	44.6 (39.0–50.9)	41.9 (37.1–47.2)	37.6 (32.6–43.3)	0.23	−15.7	*0.02*	269 (220–329)	227 (183–280)	264 (212–327)	0.75	−1.9	0.84
Conjugated	33.2 (28.8–38.3)	30.3 (26.5–34.7)	27.0 (23.1–31.5)	0.14	−18.7	*0.01*	165 (133–207)	145 (114–183)	164 (129–208)	0.96	−0.6	0.91
Unconjugated	10.2 (8.83–11.8)	10.7 (9.36–12.2)	9.92 (8.48–11.6)	0.82	−2.7	0.74	75.7 (56.4–102)	64.1 (47.8–85.9)	73.3 (53.9–99.6)	0.66	−3.2	0.82
2-Methoxyestradiol	13.7 (11.4–16.4)	13.0 (11.2–15.2)	12.3 (10.3–14.8)	0.66	−10.2	0.24	84.0 (65.4–108)	78.5 (60.2–102)	89.2 (68.5–116)	0.69	6.2	0.58
Conjugated	11.1 (9.10–13.6)	10.4 (8.71–12.3)	9.72 (7.96–11.9)	0.61	−12.4	0.17	71.3 (54.3–93.5)	68.1 (51.0–91.0)	76.0 (57.0–101)	0.81	6.6	0.58
Unconjugated	2.08 (1.76–2.45)	2.11 (1.81–2.45)	2.06 (1.75–2.43)	0.91	−1.0	0.91	9.31 (7.68–11.3)	7.48 (6.12–9.15)	8.96 (7.35–10.9)	0.62	−3.8	0.72
2-Hydroxyestrone-3-methyl ether	7.50 (6.55–8.58)	7.68 (6.75–8.73)	7.44 (6.45–8.58)	0.71	−0.8	0.92	41.4 (33.7–50.9)	36.8 (29.6–45.8)	43.1 (34.6–53.7)	0.46	4.1	0.69
4-Hydroxyestrone	7.99 (6.75–9.46)	8.46 (7.31–9.80)	7.55 (6.39–8.92)	0.90	−5.5	0.50	59.2 (47.3–74.1)	54.9 (42.2–71.4)	59.6 (47.0–75.5)	0.96	0.7	0.95
4-Methoxyestrone	4.59 (3.96–5.33)	4.24 (3.78–4.76)	3.90 (3.41–4.46)	0.21	−15.0	*0.03*	27.7 (23.0–33.4)	23.0 (18.5–28.6)	28.9 (23.5–35.6)	0.51	4.3	0.66
4-Methoxyestradiol	1.92 (1.60–2.30)	1.84 (1.59–2.13)	1.74 (1.47–2.04)	0.55	−9.4	0.22	11.3 (8.75–14.7)	10.2 (7.54–13.7)	12.4 (9.39–16.3)	0.63	9.7	0.46
16α-Hydroxyestrone	32.3 (27.1–38.4)	34.8 (29.9–40.5)	31.1 (26.1–37.1)	0.98	−3.7	0.67	241 (191–304)	214 (163–281)	233 (182–298)	0.74	−3.3	0.77
Estriol	135 (114–159)	148 (127–173)	135 (113–161)	0.51	0.0	1.00	1127 (880–1444)	1019 (751–1381)	1076 (828–1399)	0.51	−4.5	0.69
Conjugated	104 (86.7–125)	119 (100–140)	105 (86.6–128)	0.50	1.0	0.90	973 (752–1261)	887 (643–1223)	928 (705–1222)	0.44	−4.6	0.71
Unconjugated	27.4 (23.6–31.7)	26.5 (23.1–30.4)	25.6 (22.1–29.7)	0.81	−6.6	0.40	134 (108–166)	109 (83.7–141)	121 (96.7–152)	0.66	−9.7	0.30
16-Ketoestradiol	34.4 (28.9–41.0)	36.5 (31.3–42.7)	33.8 (28.3–40.5)	0.70	−1.7	0.85	279 (221–352)	252 (191–332)	265 (207–341)	0.60	−5.0	0.67
16-Epiestriol	14.5 (12.4–16.9)	16.4 (14.2–18.8)	14.8 (12.6–17.5)	0.47	2.1	0.78	83.0 (66.8–103)	77.1 (60.4–98.5)	82.5 (65.5–104)	1.00	−0.6	0.96
17-Epiestriol	11.9 (10.2–13.9)	13.7 (11.9–15.6)	12.9 (10.9–15.2)	0.30	8.4	0.30	56.4 (45.3–70.4)	49.7 (38.7–63.6)	54.9 (43.3–69.6)	0.90	−2.7	0.81

^a^Adjusted for age at blood draw (<55, 55–59, 60–64, 65–69, 70–74, 75–79 years), blood draw year (1993–1996, 1997–1998), race (white, nonwhite), smoking status (never, former, current), time since menopause (<10 years, 10–19 years, ≥20 years, missing)

^b^ *p*-trend was estimated using the Wald test for continuous height (cm)

^c^%Δ indicates the percentage change in estrogen/estrogen metabolite levels, comparing women with height ≥165 vs. <160 cm, and was estimated by taking the ratio of the geometric mean difference in estrogen/estrogen metabolite levels between women with height ≥165 vs. <160 cm to the geometric mean of women with height <160 cm, multiplied by 100

^d^ *p*-diff was estimated using the Wald test and indicates a *p* value for comparing estrogen/estrogen metabolite levels of women with height ≥165 vs. <160 cm

^e^Weighted *n* reflects weighted counts and refers to the study cohort

Note: All false discovery rate *q* values >0.05

Italized *p*-values indicate nominal statistical significance at 0.05

### Body mass index at age 18 years

Among never/former MHT users, BMI at age 18 years (≥22 vs. <20 kg/m^2^) was positively associated with estradiol (64.1 vs. 49.8 pmol/L; *p*-trend = 0.04) but was not associated with other estrogens/estrogen metabolites (Additional file 5: Table S3). The association with estradiol did not remain after adjustment for current BMI (data not shown), suggesting that the association may be accounted for by current BMI. Among current MHT users, BMI at age 18 years was not associated with estrogens/estrogen metabolites (Additional file 5: Table S3). There was no statistically significant interaction between BMI at age 18 years and current BMI (*p*-interaction >0.05) (data not shown).

Most associations remained at 5% FDR (Tables 2, 3, 4 and 5, Additional file 1: Table S1, Additional file 4: Table S2, Additional file 5: Table S3). Results were similar after excluding endometrial or ovarian cancer cases at the time of last follow-up (Additional file 6: Table S4), after excluding women who reported a history of diabetes at baseline (data not shown), after excluding women with low BMI (<18.5 kg/m^2^) (data not shown), after excluding women using any potential hormone-related medications (data not shown), and after further stratifying by never vs. former MHT users (data not shown). Results also did not change after excluding outliers (data not shown).

## Discussion

To the best of our knowledge, this study is the first to examine the relationships of anthropometric measures with detailed serum estrogen/estrogen metabolite measures in postmenopausal women by MHT use. In this cross-sectional analysis, current measured BMI was positively associated with parent estrogens and all estrogen metabolites evaluated among never/former MHT users. Adjusted for unconjugated estradiol, current BMI was inversely associated with methylated 2-catechols but not with the other estrogen metabolites measured, suggesting that associations with individual estrogen metabolites among never/former MHT users were driven largely by their correlations with unconjugated estradiol. Associations with measured WHR were not independent of current BMI, suggesting that the associations were driven by overall adiposity rather than by fat distribution per se. BMI at age 18 years and height were not associated with postmenopausal estrogen/estrogen metabolite levels.

Our findings of positive associations between current BMI and parent estrogens are consistent with those from previous studies [8, 21–23, 32–34]. A pooled analysis of eight studies estimated 83% higher levels of circulating total estradiol and 60% higher levels of total estrone in postmenopausal obese women (BMI ≥30 kg/m^2^) compared with lean women (BMI <22.5 kg/m^2^) [8]. These findings are also in line with biological evidence that supports the major source of estrogens in postmenopausal women being via aromatization of androgens in adipose tissue [35, 36]. Further, although few studies have examined the associations in current MHT users, our findings are consistent with data from the Nurses’ Health Study regarding unconjugated estradiol BMI (≥30 vs. <25 kg/m^2^) associations in both never/former and current MHT users and the finding of stronger positive associations in never/former users (62% vs. 22% change in estradiol levels) [23]. In the present study, unconjugated estradiol was consistently positively associated with current BMI (≥30 vs. <25 kg/m^2^) among both never/former (130% change) and current MHT users (20% change), although the association was not statistically significant in current MHT users. The differential associations between BMI and parent estrogens by MHT use mirror the similar differential associations of BMI with cancer risk [37, 38], further supporting the notion that endogenous estrogens may mediate the BMI-cancer association, although alternative mechanisms may also exist [24, 39, 40]. The positive associations of BMI with postmenopausal breast cancer [37, 38] and endometrial cancer [41, 42] risk have been found consistently in women not using MHT; however, the associations have been weakly positive or near null among current MHT users [37, 38, 41, 42], possibly owing to a threshold effect of circulating estrogens in current MHT users. Current MHT users have higher circulating levels of estrogens; thus, increased estrogen production by adipose tissue may not contribute to further increase in cancer risk. Moreover, given the high estrogen levels in current MHT users, the association with BMI on the relative scale may be masked, whereas an absolute difference in estrogen concentration may be apparent, as in never/former users.

To date, few studies have examined current BMI in relation to estrogen metabolism beyond parent estrogens. Earlier studies measured only two estrogen metabolites that have been thought to be the most and the least carcinogenic: 16α-hydroxyestrone and 2-hydroxyestrone, respectively [43–46]. These earlier studies have shown an inverse association between adiposity and the ratio of urinary 2-hydroxyestrone to 16α-hydroxyestrone in both pre- and postmenopausal women [44, 45, 47]. In a more recent analysis in the Prostate, Lung, Colorectal, and Ovarian Cancer Screening Trial cohort, self-reported BMI was positively correlated with all 15 serum estrogens/estrogen metabolites among postmenopausal women who had never used MHT [34]; however, whether the associations with estrogen metabolites remained after accounting for correlations with parent estrogens was not assessed. Although we observed similar positive associations with estrogens/estrogen metabolites using measured BMI among never/former MHT users in the present study, the associations did not remain after adjustment for unconjugated estradiol. Similarly to our findings of inverse associations between BMI and methylated 2-catechols after adjusting for unconjugated estradiol in postmenopausal women, researchers in a study of premenopausal women found that BMI was inversely associated with most methylated catechols measured in urine [48]. In our secondary analyses, we also observed that obese women appeared to be less likely to metabolize parent estrogens into child metabolites in general but more likely to favor metabolism of parent estrogens into 16-pathway estrogen metabolites over 2- or 4-methylated catechols. Our findings provide novel, additional detailed information about patterns of estrogen metabolism. They are in line with results from a previous study that evaluated 12 estrogens/estrogen metabolites in prepubertal girls and observed higher levels of 16α-hydroxyestrone and lower levels of 2-methoxyestradiol in obese girls (BMI >95th percentile vs. 10th–85th percentile) [49]. Although the exact mechanisms for this relationship are not clear, reduced catechol-*O*-methyltransferase (COMT) activity associated with obesity [50] and/or suppressed COMT activity by estradiol [51, 52] may explain the decreased methylation of catechols with increased BMI observed in the present study. Methylation prevents catechols from metabolizing into quinones, which can form quinone adducts and induce oxidative DNA damage [53]. Further, reduced levels of methylated 2-catechols in obese women (BMI ≥30 vs. <25 kg/m^2^) are also in line with studies that have consistently shown lower ratios of 2-pathway metabolites to parent estrogens to be associated with higher postmenopausal breast cancer risk [17–19].

In the present study, we did not observe associations of WHR with estrogens/estrogen metabolites after adjustment for current BMI. Similarly, in studies where body fat distribution was measured by dual-energy X-ray absorptiometry [32] or measured WHR [33], central obesity was not associated with circulating unconjugated estradiol independent of BMI among postmenopausal women not using MHT, suggesting that body fat distribution does not provide additional information about circulating estradiol beyond what overall adiposity (as measured by BMI) provides.

In the present study, adult height and BMI at age 18 years were not associated with estrogens/estrogen metabolites after adjustment for current BMI. BMI at age 18 years and height may indicate early-life nutritional status, which may not influence estrogen metabolism in postmenopausal women. In a study of premenopausal women, BMI at age 18 years was not associated with urinary estrogen/estrogen metabolite levels; however, contrary to our findings, height was inversely associated with urinary parent estrogens and 2-pathway metabolites in the same study [48]. The difference in results may be due to the variation in study populations (e.g., age, menopausal status) or biospecimen used (circulating vs. excreted levels).

We acknowledge several limitations of this study. We measured circulating estrogens/estrogen metabolites in a single baseline serum sample. However, in a previous study using the same assay we used, researchers showed moderate to high 1-year ICCs in postmenopausal women for parent estrogens (0.72 for estrone, 0.65 for estradiol) and most estrogen metabolites of 2-, 4-, and 16-pathways (range 0.35–0.53), with the exception of 2-methoxyestrone (0.10) and 2-hydroxyestrone-3-methyl ether (0.14) [17], suggesting that measured serum estrogens/estrogen metabolites may also adequately represent postmenopausal levels over at least 1 year. BMI at age 18 years was based on self-reported height and weight. However, measurement error in this context is unlikely to be related to serum estrogen levels and, if present, would likely underestimate the associations. Although we cannot exclude the possibility of false-positives, most associations with current BMI remained at 5% FDR.

Despite these limitations, this study has important strengths. Whereas most epidemiologic studies have used self-reported measures, measurement error in the present study was reduced by using measured height, weight, and waist and hip circumferences. Use of a high-performance LC-MS/MS assay also allowed comprehensive evaluation of individual estrogens/estrogen metabolites with high reliability, sensitivity, and specificity. By stratifying the analysis by MHT use, we were able to take into account variations in estrogen/estrogen metabolite levels by exogenous hormone use. Further, use of a large sample size and careful adjustment for potential confounders assessed at blood collection increased the validity of the results.

## Conclusions

In this comprehensive analysis of measured anthropometrics and serum estrogens/estrogen metabolites, we observed strong positive associations between current BMI and parent estrogens in postmenopausal women not using MHT. Adjusted for unconjugated estradiol, current BMI was also associated with lower levels of methylated catechols among both never/former and current MHT users. Similar estrogen metabolism patterns have previously been associated with higher breast cancer risk; thus, these findings further support the need to prospectively evaluate the roles of endogenous estrogens/estrogen metabolites in the BMI-breast cancer risk association.

## Additional files

Additional file 1: Table S1. Geometric means (pmol/L) and 95% CIs of serum estrogens/estrogen metabolites by current BMI (<25, 25–29.9, 30–34.9, ≥40 kg/m^2^) in postmenopausal women not using menopausal hormone therapy in the Women’s Health Initiative Observational Study. (PDF 93 kb)
Additional file 2: Figure S1. Percentages of parent estrogens (estradiol and estrone) and child estrogen metabolites (2-, 4-, and 16-hydroxylation pathway metabolites) out of summed estrogens/estrogen metabolites by current BMI among current menopausal hormone therapy users. (PPTX 63 kb)
Additional file 3: Figure S2. Percentages of each pathway estrogens/estrogen metabolites (2-catechols, methylated 2-catechols, 4-catechols, methylated 4-catechols, 16-pathway metabolites) out of summed child estrogen metabolites by current BMI among current menopausal hormone therapy users. (PPTX 70 kb)
Additional file 4: Table S2. Geometric means (pmol/L) and 95% CIs of serum estrogens/estrogen metabolites by waist-to-hip ratio in postmenopausal women currently using menopausal hormone therapy in the Women’s Health Initiative Observational Study. (PDF 95 kb)
Additional file 5: Table S3. Geometric means (pmol/L) and 95% CIs of serum estrogens/estrogen metabolites by BMI at age 18 years in postmenopausal women in the Women’s Health Initiative Observational Study. (PDF 94 kb)
Additional file 6: Table S4. Geometric means (pmol/L) and 95% CIs of serum estrogens/estrogen metabolites by current BMI in postmenopausal women not using menopausal hormone therapy among control subjects only in the Women’s Health Initiative Observational Study. (PDF 103 kb)
Additional file 7: Women’s Health Initiative clinical centers. (DOCX 13 kb)

## Abbreviations

BMI: Body mass index
COMT: Catechol-*O*-methyltransferase
FDR: False discovery rate
GM: Geometric mean
ICC: Intraclass correlation coefficient
LC-MS/MS: Liquid chromatography-tandem mass spectrometry
MET: Metabolic equivalent of task
MHT: Menopausal hormone therapy
OS: Observational Study
WHI: Women’s Health Initiative
WHR: Waist-to-hip ratio

## References

[1] Aune D, Navarro Rosenblatt DA, Chan DS, Vingeliene S, Abar L, Vieira AR (2015). Anthropometric factors and endometrial cancer risk: a systematic review and dose-response meta-analysis of prospective studies. Ann Oncol.

[2] Cheraghi Z, Poorolajal J, Hashem T, Esmailnasab N, Doosti IA (2012). Effect of body mass index on breast cancer during premenopausal and postmenopausal periods: a meta-analysis. PLoS One.

[3] Collaborative Group on Epidemiological Studies of Ovarian Cancer (2012). Ovarian cancer and body size: individual participant meta-analysis including 25,157 women with ovarian cancer from 47 epidemiological studies. PLoS Med.

[4] Connolly BS, Barnett C, Vogt KN, Li T, Stone J, Boyd NF (2002). A meta-analysis of published literature on waist-to-hip ratio and risk of breast cancer. Nutr Cancer.

[5] van den Brandt PA, Spiegelman D, Yaun SS, Adami HO, Beeson L, Folsom AR (2000). Pooled analysis of prospective cohort studies on height, weight, and breast cancer risk. Am J Epidemiol.

[6] Renehan AG, Tyson M, Egger M, Heller RF, Zwahlen M (2008). Body-mass index and incidence of cancer: a systematic review and meta-analysis of prospective observational studies. Lancet.

[7] Neuhouser ML, Aragaki AK, Prentice RL, Manson JE, Chlebowski R, Carty CL (2015). Overweight, obesity, and postmenopausal invasive breast cancer risk: a secondary analysis of the Women’s Health Initiative randomized clinical trials. JAMA Oncol.

[8] Key TJ, Appleby PN, Reeves GK, Roddam A, Dorgan JF, Longcope C (2003). Body mass index, serum sex hormones, and breast cancer risk in postmenopausal women. J Natl Cancer Inst.

[9] Siiteri PK (1987). Adipose tissue as a source of hormones. Am J Clin Nutr.

[10] Schroeder DG, Martorell R, Rivera JA, Ruel MT, Habicht JP (1995). Age differences in the impact of nutritional supplementation on growth. J Nutr.

[11] Key T, Appleby P, Barnes I, Reeves G, Endogenous Hormones and Breast Cancer Collaborative Group (2002). Endogenous sex hormones and breast cancer in postmenopausal women: reanalysis of nine prospective studies. J Natl Cancer Inst.

[12] Brown SB, Hankinson SE (2015). Endogenous estrogens and the risk of breast, endometrial, and ovarian cancers. Steroids.

[13] Santen RJ, Yue W, Wang JP (2015). Estrogen metabolites and breast cancer. Steroids.

[14] Cavalieri E, Frenkel K, Liehr JG, Rogan E, Roy D (2000). Estrogens as endogenous genotoxic agents—DNA adducts and mutations. J Natl Cancer Inst Monogr.

[15] Cavalieri E, Chakravarti D, Guttenplan J, Hart E, Ingle J, Jankowiak R (2006). Catechol estrogen quinones as initiators of breast and other human cancers: implications for biomarkers of susceptibility and cancer prevention. Biochim Biophys Acta.

[16] Yager JD, Davidson NE (2006). Estrogen carcinogenesis in breast cancer. N Engl J Med.

[17] Falk RT, Brinton LA, Dorgan JF, Fuhrman BJ, Veenstra TD, Xu X (2013). Relationship of serum estrogens and estrogen metabolites to postmenopausal breast cancer risk: a nested case-control study. Breast Cancer Res.

[18] Dallal CM, Tice JA, Buist DS, Bauer DC, Lacey JV, Cauley JA (2014). Estrogen metabolism and breast cancer risk among postmenopausal women: a case-cohort study within B ~ FIT. Carcinogenesis.

[19] Fuhrman BJ, Schairer C, Gail MH, Boyd-Morin J, Xu X, Sue LY (2012). Estrogen metabolism and risk of breast cancer in postmenopausal women. J Natl Cancer Inst.

[20] Brinton LA, Trabert B, Anderson GL, Falk RT, Felix AS, Fuhrman BJ (2016). Serum estrogens and estrogen metabolites and endometrial cancer risk among postmenopausal women. Cancer Epidemiol Biomarkers Prev.

[21] Hankinson SE, Willett WC, Manson JE, Hunter DJ, Colditz GA, Stampfer MJ (1995). Alcohol, height, and adiposity in relation to estrogen and prolactin levels in postmenopausal women. J Natl Cancer Inst.

[22] Lukanova A, Lundin E, Zeleniuch-Jacquotte A, Muti P, Mure A, Rinaldi S (2004). Body mass index, circulating levels of sex-steroid hormones, IGF-I and IGF-binding protein-3: a cross-sectional study in healthy women. Eur J Endocrinol.

[23] Tworoger SS, Missmer SA, Barbieri RL, Willett WC, Colditz GA, Hankinson SE (2005). Plasma sex hormone concentrations and subsequent risk of breast cancer among women using postmenopausal hormones. J Natl Cancer Inst.

[24] Edlefsen KL, Jackson RD, Prentice RL, Janssen I, Rajkovic A, O’Sullivan MJ (2010). The effects of postmenopausal hormone therapy on serum estrogen, progesterone, and sex hormone-binding globulin levels in healthy postmenopausal women. Menopause.

[25] Nachtigall LE, Raju U, Banerjee S, Wan L, Levitz M (2000). Serum estradiol-binding profiles in postmenopausal women undergoing three common estrogen replacement therapies: associations with sex hormone-binding globulin, estradiol, and estrone levels. Menopause.

[26] Trabert B, Brinton LA, Anderson GL, Pfeiffer RM, Falk RT, Strickler HD (2016). Circulating estrogens and postmenopausal ovarian cancer risk in the Women’s Health Initiative Observational Study. Cancer Epidemiol Biomarkers Prev.

[27] Langer RD, White E, Lewis CE, Kotchen JM, Hendrix SL, Trevisan M (2003). The Women’s Health Initiative Observational Study: baseline characteristics of participants and reliability of baseline measures. Ann Epidemiol.

[28] The Women’s Health Initiative Study Group (1998). Design of the Women’s Health Initiative clinical trial and observational study. Control Clin Trials.

[29] World Health Organization (WHO) (1995). Physical status: the use and interpretation of anthropometry. Report of a WHO expert committee. Technical Report Series number 854.

[30] Li HL, Gail MH (2012). Efficient adaptively weighted analysis of secondary phenotypes in case-control genome-wide association studies. Hum Hered.

[31] Rosner B (1983). Percentage points for a generalized ESD many-outlier procedure. Technometrics.

[32] Mahabir S, Baer DJ, Johnson LL, Hartman TJ, Dorgan JF, Campbell WS (2006). Usefulness of body mass index as a sufficient adiposity measurement for sex hormone concentration associations in postmenopausal women. Cancer Epidemiol Biomarkers Prev.

[33] Liedtke S, Schmidt ME, Vrieling A, Lukanova A, Becker S, Kaaks R (2012). Postmenopausal sex hormones in relation to body fat distribution. Obesity (Silver Spring).

[34] Schairer C, Fuhrman BJ, Boyd-Morin J, Genkinger JM, Gail MH, Hoover RN (2016). Quantifying the role of circulating unconjugated estradiol in mediating the body mass index-breast cancer association. Cancer Epidemiol Biomarkers Prev.

[35] Folkerd EJ, James VH (1983). Aromatization of steroids in peripheral tissues. J Steroid Biochem.

[36] Grodin JM, Siiteri PK, MacDonald PC (1973). Source of estrogen production in postmenopausal women. J Clin Endocrinol Metab.

[37] White KK, Park SY, Kolonel LN, Henderson BE, Wilkens LR (2012). Body size and breast cancer risk: the Multiethnic Cohort. Int J Cancer.

[38] Huang Z, Hankinson SE, Colditz GA, Stampfer MJ, Hunter DJ, Manson JE (1997). Dual effects of weight and weight gain on breast cancer risk. JAMA.

[39] Gunter MJ, Hoover DR, Yu H, Wassertheil-Smoller S, Rohan TE, Manson JE (2009). Insulin, insulin-like growth factor-I, and risk of breast cancer in postmenopausal women. J Natl Cancer Inst.

[40] Hvidtfeldt UA, Gunter MJ, Lange T, Chlebowski RT, Lane D, Farhat GN (2012). Quantifying mediating effects of endogenous estrogen and insulin in the relation between obesity, alcohol consumption, and breast cancer. Cancer Epidemiol Biomarkers Prev.

[41] Chang SC, Lacey JV, Brinton LA, Hartge P, Adams K, Mouw T (2007). Lifetime weight history and endometrial cancer risk by type of menopausal hormone use in the NIH-AARP diet and health study. Cancer Epidemiol Biomarkers Prev.

[42] Dougan MM, Hankinson SE, Vivo ID, Tworoger SS, Glynn RJ, Michels KB (2015). Prospective study of body size throughout the life-course and the incidence of endometrial cancer among premenopausal and postmenopausal women. Int J Cancer.

[43] Matthews CE, Fowke JH, Dai Q, Leon Bradlow H, Jin F, Shu XO (2004). Physical activity, body size, and estrogen metabolism in women. Cancer Causes Control.

[44] Coker AL, Crane MM, Sticca RP, Sepkovic DW (1997). Re: Ethnic differences in estrogen metabolism in healthy women. J Natl Cancer Inst.

[45] Schneider J, Bradlow HL, Strain G, Levin J, Anderson K, Fishman J (1983). Effects of obesity on estradiol metabolism: decreased formation of nonuterotropic metabolites. J Clin Endocrinol Metab.

[46] Modugno F, Kip KE, Cochrane B, Kuller L, Klug TL, Rohan TE (2006). Obesity, hormone therapy, estrogen metabolism and risk of postmenopausal breast cancer. Int J Cancer.

[47] Fishman J, Boyar RM, Hellman L (1975). Influence of body weight on estradiol metabolism in young women. J Clin Endocrinol Metab.

[48] Xie J, Eliassen AH, Xu X, Matthews CE, Hankinson SE, Ziegler RG (2012). Body size in relation to urinary estrogens and estrogen metabolites (EM) among premenopausal women during the luteal phase. Hormones Cancer.

[49] Mauras N, Santen RJ, Colon-Otero G, Hossain J, Wang Q, Mesaros C (2015). Estrogens and their genotoxic metabolites are increased in obese prepubertal girls. J Clin Endocrinol Metab.

[50] Wang SS, Morton LM, Bergen AW, Lan EZ, Chatterjee N, Kvale P (2007). Genetic variation in catechol-*O*-methyltransferase (COMT) and obesity in the prostate, lung, colorectal, and ovarian (PLCO) cancer screening trial. Hum Genet.

[51] Jiang H, Xie T, Ramsden DB, Ho SL (2003). Human catechol-*O*-methyltransferase down-regulation by estradiol. Neuropharmacology.

[52] Xie T, Ho SL, Ramsden D (1999). Characterization and implications of estrogenic down-regulation of human catechol-*O*-methyltransferase gene transcription. Mol Pharmacol.

[53] Yager JD (2000). Endogenous estrogens as carcinogens through metabolic activation. J Natl Cancer Inst Monogr.

