# Supplementary material for: Anthropometric measures and serum estrogen metabolism in postmenopausal women: the Women’s Health Initiative Observational Study
Source: Breast Cancer Res. 2017 Mar 11;19:28. doi: 10.1186/s13058-017-0810-0 (PMC5346241; doi:10.1186/s13058-017-0810-0)
Supplement: Additional file 1: Table S1. — Geometric means (pmol/L) and 95% CIs of serum estrogens/estrogen metabolites by current BMI (<25, 25–29.9, 30–34.9, ≥40 kg/m2) in postmenopausal women not using menopausal hormone therapy in the Women’s Health Initiative Observational Study. (PDF 93 kb) [file 13058_2017_810_MOESM1_ESM.pdf]

Table S1. Geometric means (pmol/L) and 95% confidence intervals (CI) of serum estrogens/estrogen metabolites by current BMI (<25, 25-29.9, 30-34.9, 35-39.9, ≥40 kg/m^2) in postmenopausal women not using menopausal hormone therapy: the Women's Health Initiative Observational Study

	**Model 1**[a]						**Model 1 + unconjugated estradiol** [b]					
	**Geometric means (95% CI)**						**Geometric means (95% CI)**					
**Current BMI (kg/m^2)**	**<25**	**25-29.9**	**30-34.9**	**35-39.9**	**≥40**	**p-trend**[c]	**<25**	**25-29.9**	**30-34.9**	**35-39.9**	**≥40**	**p-trend**[c]
**Median (kg/m^2)**	22.7	27.3	31.8	37.0	44.0		22.7	27.3	31.8	37.0	44.0	
**N**	345	294	168	85	61		345	294	168	85	61	
**Weighted N**[d]	13571	9351	5205	1756	931		13571	9351	5205	1756	931	
**Estrone**	236	335	386	518	593	**<0.001****	314	344	328	327	312	0.91
Conjugated	177	264	309	423	479	**<0.001****	237	271	261	263	246	0.59
Unconjugated	50.8	62.1	69.6	84.0	100	**<0.001****	64.5	63.4	60.8	57.2	58.5	0.13
**Estradiol**	45.1	58.5	63.8	87.5	126	**<0.001****	60.4	60.1	54.1	54.7	65	0.33
Conjugated	30.5	39.8	39.7	53.9	78.5	**<0.001****	38.1	40.6	34.9	37.5	47.2	0.99
Unconjugated	9.97	14.8	19.6	30.8	40.8	**<0.001****	NA	NA	NA	NA	NA	NA
**2-Hydroxyestrone**	55.5	71.6	81.7	81.2	92.8	**<0.001****	66.7	72.8	73.6	60.3	61.1	0.83
**2-Hydroxyestrdaiol**	14.2	17.7	20	20.2	23.3	**<0.001****	16.9	18	18.1	15.2	15.7	0.69
**2-Methoxyestrone**	37.7	43.6	46.2	49.4	53.4	**0.001****	44.9	44.3	41.8	37.2	35.9	0.05
Conjugated	27.3	32.1	33.5	34.8	40.6	**0.002****	31.9	32.5	30.7	27.1	28.7	0.25
Unconjugated	9.64	10.5	10.9	13.9	12.6	**0.02***	12.1	10.7	9.53	9.61	7.48	**<0.001****
**2-Methoxyestradiol**	11.3	13.5	15.4	19.1	18.4	**<0.001****	13.8	13.7	13.8	13.9	11.8	0.50
Conjugated	8.98	10.7	12.8	15.2	14.2	**<0.001****	10.8	10.9	11.5	11.2	9.3	0.83
Unconjugated	1.87	2.19	2.03	3.05	3.53	**<0.001****	2.35	2.24	1.79	2.12	2.12	**0.002***
**2-Hydroxyestrone-3-methyl ether**	6.74	7.94	8.59	9.18	8.66	**0.004****	7.74	8.04	7.94	7.33	6.32	0.24
**4-Hydroxyestrone**	6.75	8.68	10.1	9.88	11.2	**<0.001****	8.05	8.82	9.13	7.43	7.51	0.96
**4-Methoxyestrone**	3.93	4.36	4.86	5.00	4.60	**0.02***	4.52	4.42	4.49	3.99	3.36	0.19
**4-Methoxyestradiol**	1.65	1.86	2.10	2.50	2.47	**<0.001****	1.96	1.89	1.90	1.89	1.66	0.30
**16α-Hydroxyestrone**	27.4	35.8	40.9	41.4	46	**<0.001****	33.1	36.4	36.8	30.6	30.1	0.92
**Estriol**	113	155	171	192	198	**<0.001****	138	158	153	139	126	0.94
Conjugated estriol	86.7	124	137	153	156	**<0.001****	106	126	123	111	99.6	0.60
Unconjugated	23	27.3	31	36.8	38.9	**<0.001****	28.0	27.8	27.7	26.8	24.9	0.29
**16-Ketoestradiol**	29	38.2	43.3	44.4	50.4	**<0.001****	34.9	38.8	39	32.8	33	0.95
**16-Epiestriol**	12.8	16.4	18.7	20.0	21.4	**<0.001****	15.2	16.6	17.0	15.2	14.6	0.78
**17-Epiestriol**	10.7	13.5	16.1	16.5	18.4	**<0.001****	12.7	13.7	14.6	12.5	12.5	0.72

[a] Model 1: Adjusted for age at blood draw (<55, 55-59, 60-64, 66-69, 70-74, 75-79 years), blood draw year (1993-1996, 1997-1998), race (white, non-white), smoking status (never, former, current), time since menopause (<10, 10-19, ≥20 years, missing), physical activity (0, 0.1-9.9, ≥10 MET-hr/wk).
[b] Model 1 + unconjugated estradiol (log-transformed, continuous).
[c] p-trend was estimated using the Wald test for continuous BMI (kg/m^2).
[d] Weighted N reflects weighted counts and refer to the study cohort.
* indicates False Discovery Rate (FDR) q-value<0.05 and ≥0.01. ** indicates FDR q-value<0.01.
Abbreviations: BMI=body mass index, CI=confidence interval, NA=not applicable.
