# Supplementary material for: Anthropometric measures and serum estrogen metabolism in postmenopausal women: the Women’s Health Initiative Observational Study
Source: Breast Cancer Res. 2017 Mar 11;19:28. doi: 10.1186/s13058-017-0810-0 (PMC5346241; doi:10.1186/s13058-017-0810-0)
Supplement: Additional file 2: Figure S1. — Percentages of parent estrogens (estradiol and estrone) and child estrogen metabolites (2-, 4-, and 16-hydroxylation pathway metabolites) out of summed estrogens/estrogen metabolites by current BMI among current menopausal hormone therapy users. (PPTX 63 kb) [file 13058_2017_810_MOESM2_ESM.pptx]

## Slide 1
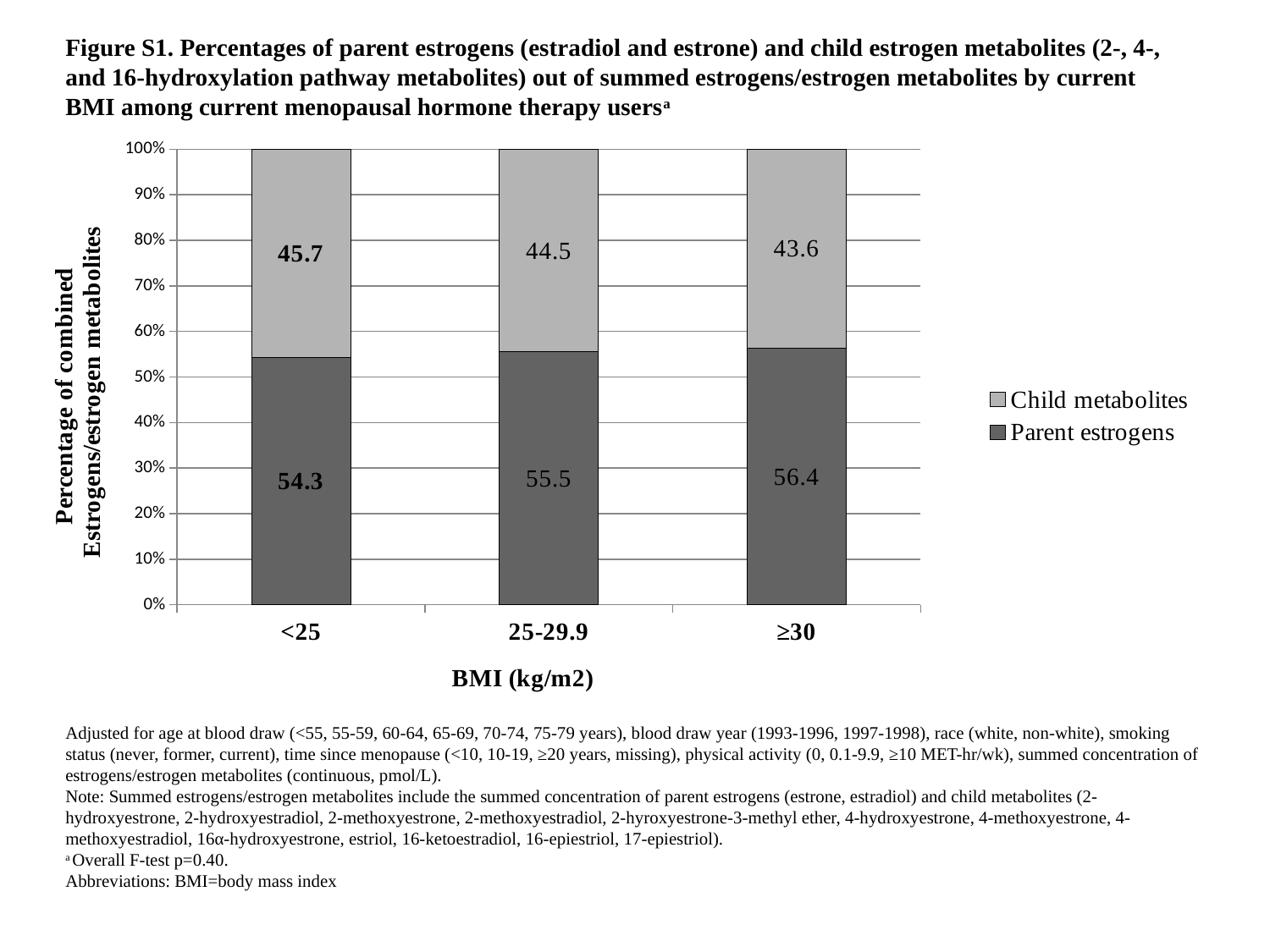

# Figure S1. Percentages of parent estrogens (estradiol and estrone) and child estrogen metabolites (2-, 4-, and 16-hydroxylation pathway metabolites) out of summed estrogens/estrogen metabolites by current BMI among current menopausal hormone therapy usersa
### Chart
| Category | Parent estrogens | Child metabolites |
|---|---|---|
| <25 | 54.3 | 45.7 |
| 25-29.9 | 55.5 | 44.5 |
| ≥30 | 56.4 | 43.6 |Adjusted for age at blood draw (<55, 55-59, 60-64, 65-69, 70-74, 75-79 years), blood draw year (1993-1996, 1997-1998), race (white, non-white), smoking status (never, former, current), time since menopause (<10, 10-19, ≥20 years, missing), physical activity (0, 0.1-9.9, ≥10 MET-hr/wk), summed concentration of estrogens/estrogen metabolites (continuous, pmol/L).
Note: Summed estrogens/estrogen metabolites include the summed concentration of parent estrogens (estrone, estradiol) and child metabolites (2-hydroxyestrone, 2-hydroxyestradiol, 2-methoxyestrone, 2-methoxyestradiol, 2-hyroxyestrone-3-methyl ether, 4-hydroxyestrone, 4-methoxyestrone, 4-methoxyestradiol, 16α-hydroxyestrone, estriol, 16-ketoestradiol, 16-epiestriol, 17-epiestriol).
a Overall F-test p=0.40.
Abbreviations: BMI=body mass index
