# Supplementary material for: Anthropometric measures and serum estrogen metabolism in postmenopausal women: the Women’s Health Initiative Observational Study
Source: Breast Cancer Res. 2017 Mar 11;19:28. doi: 10.1186/s13058-017-0810-0 (PMC5346241; doi:10.1186/s13058-017-0810-0)
Supplement: Additional file 3: Figure S2. — Percentages of each pathway estrogens/estrogen metabolites (2-catechols, methylated 2-catechols, 4-catechols, methylated 4-catechols, 16-pathway metabolites) out of summed child estrogen metabolites by current BMI among current menopausal hormone therapy users. (PPTX 70 kb) [file 13058_2017_810_MOESM3_ESM.pptx]

## Slide 1
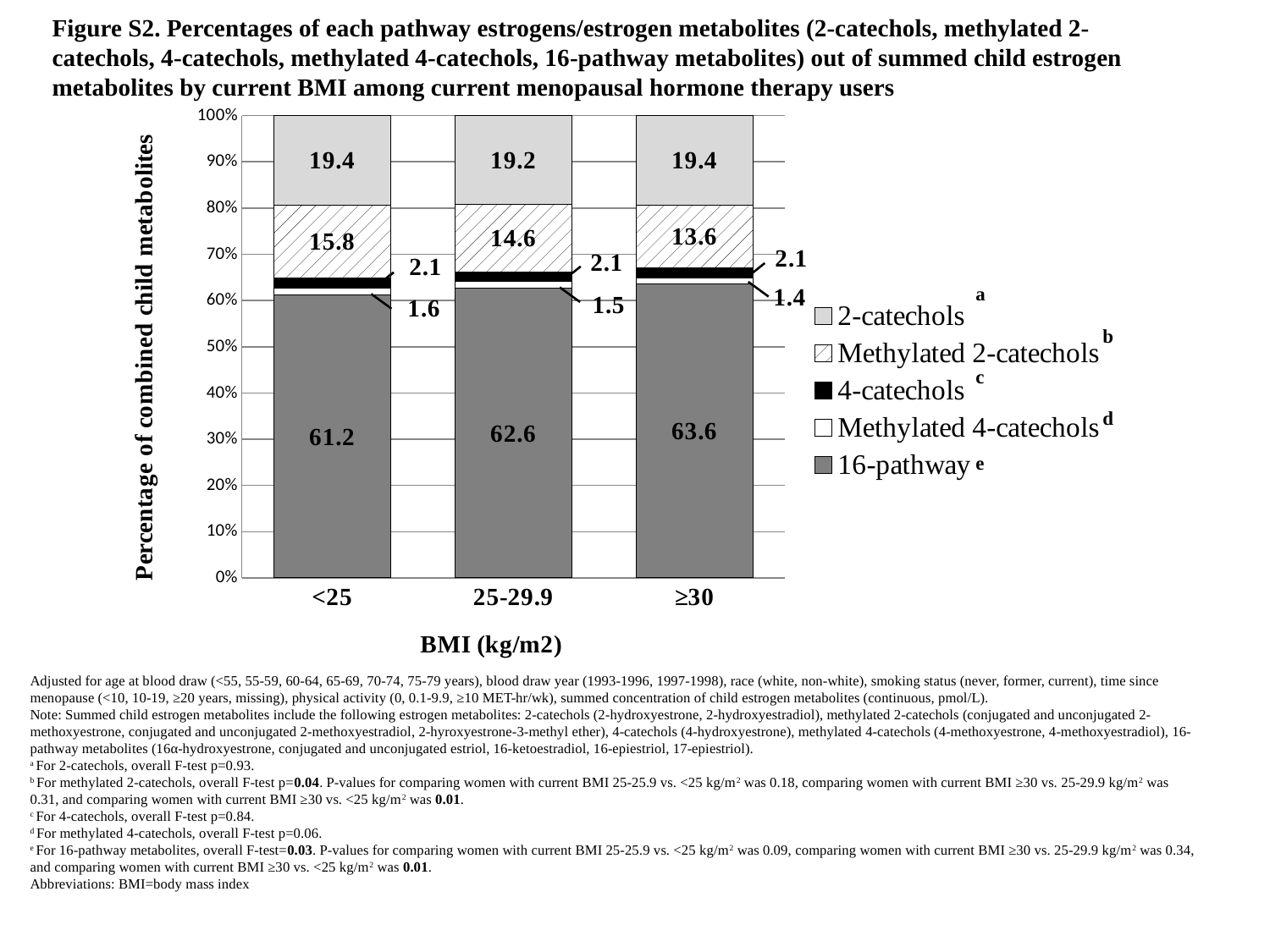

Figure S2. Percentages of each pathway estrogens/estrogen metabolites (2-catechols, methylated 2-catechols, 4-catechols, methylated 4-catechols, 16-pathway metabolites) out of summed child estrogen metabolites by current BMI among current menopausal hormone therapy users
### Chart
| Category | 16-pathway | Methylated 4-catechols | 4-catechols | Methylated 2-catechols | 2-catechols |
|---|---|---|---|---|---|
| <25 | 61.2 | 1.6 | 2.1 | 15.8 | 19.4 |
| 25-29.9 | 62.6 | 1.5 | 2.1 | 14.6 | 19.2 |
| ≥30 | 63.6 | 1.4 | 2.1 | 13.6 | 19.4 |a
b
c
d
e
Adjusted for age at blood draw (<55, 55-59, 60-64, 65-69, 70-74, 75-79 years), blood draw year (1993-1996, 1997-1998), race (white, non-white), smoking status (never, former, current), time since menopause (<10, 10-19, ≥20 years, missing), physical activity (0, 0.1-9.9, ≥10 MET-hr/wk), summed concentration of child estrogen metabolites (continuous, pmol/L).
Note: Summed child estrogen metabolites include the following estrogen metabolites: 2-catechols (2-hydroxyestrone, 2-hydroxyestradiol), methylated 2-catechols (conjugated and unconjugated 2-methoxyestrone, conjugated and unconjugated 2-methoxyestradiol, 2-hyroxyestrone-3-methyl ether), 4-catechols (4-hydroxyestrone), methylated 4-catechols (4-methoxyestrone, 4-methoxyestradiol), 16-pathway metabolites (16α-hydroxyestrone, conjugated and unconjugated estriol, 16-ketoestradiol, 16-epiestriol, 17-epiestriol).
a For 2-catechols, overall F-test p=0.93.
b For methylated 2-catechols, overall F-test p=0.04. P-values for comparing women with current BMI 25-25.9 vs. <25 kg/m2 was 0.18, comparing women with current BMI ≥30 vs. 25-29.9 kg/m2 was 0.31, and comparing women with current BMI ≥30 vs. <25 kg/m2 was 0.01.
c For 4-catechols, overall F-test p=0.84.
d For methylated 4-catechols, overall F-test p=0.06.
e For 16-pathway metabolites, overall F-test=0.03. P-values for comparing women with current BMI 25-25.9 vs. <25 kg/m2 was 0.09, comparing women with current BMI ≥30 vs. 25-29.9 kg/m2 was 0.34, and comparing women with current BMI ≥30 vs. <25 kg/m2 was 0.01.
Abbreviations: BMI=body mass index
