# Supplementary material for: Anthropometric measures and serum estrogen metabolism in postmenopausal women: the Women’s Health Initiative Observational Study
Source: Breast Cancer Res. 2017 Mar 11;19:28. doi: 10.1186/s13058-017-0810-0 (PMC5346241; doi:10.1186/s13058-017-0810-0)
Supplement: Additional file 4: Table S2. — Geometric means (pmol/L) and 95% CIs of serum estrogens/estrogen metabolites by waist-to-hip ratio in postmenopausal women currently using menopausal hormone therapy in the Women’s Health Initiative Observational Study. (PDF 95 kb) [file 13058_2017_810_MOESM4_ESM.pdf]

Table S2. Geometric means (pmol/L) and 95% confidence intervals (CI) of serum estrogens/estrogen metabolites by waist to hip ratio in postmenopausal women currently using menopausal hormone therapy: the Women's Health Initiative Observational Study

	Model 1[a]						Model 1 + Current BMI [b]					
	Geometric means (95% CI)			p-trend[c]	%Δ[d]	p-diff[e]	Geometric means (95% CI)			p-trend[c]	%Δ[d]	p-diff[e]
	**<0.76**	**0.76-0.82**	**≥0.83**				**<0.76**	**0.76-0.82**	**≥0.83**			
**Median**	0.73	0.79	0.88				0.73	0.79	0.88			
**N**	341	321	215				341	321	215			
**Weighted N[f]**	9444	8370	7338				9444	8370	7338			
**Estrone**	3038 (2213,	3407 (2588,	2761 (2061, 3699)	0.32	-9.1	0.50	3001 (2175, 4141)	3386 (2567, 4467)	2792 (2081, 3746)	0.44	-7.0	0.63
Conjugated	2759 (1988,	3165 (2386,	2499 (1850, 3376)	0.32	-9.4	0.51	2718 (1948, 3794)	3144 (2365, 4181)	2529 (1872, 3417)	0.44	-7.0	0.64
Unconjugated	224 (178, 283)	225 (183, 276)	194 (154, 245)	0.18	-13.4	0.20	219 (173, 277)	223 (181, 274)	198 (157, 250)	0.36	-9.6	0.38
**Estradiol**	433 (321, 584)	459 (346, 610)	386 (293, 507)	0.30	-10.9	0.38	418 (308, 566)	451 (339, 600)	398 (303, 524)	0.62	-4.8	0.73
Conjugated	357 (256, 499)	395 (289, 540)	308 (229, 416)	0.19	-13.7	0.32	342 (244, 481)	387 (283, 530)	319 (237, 430)	0.43	-6.7	0.66
Unconjugated	40.8 (31.2, 53.4)	43.7 (34.9, 54.6)	47.3 (37.1, 60.2)	0.11	15.9	0.21	42.2 (32.1, 55.4)	44.3 (35.3, 55.7)	46.0 (36.2, 58.4)	0.24	9.0	0.47
**2-Hydroxyestrone**	435 (345, 548)	480 (392, 587)	389 (310, 490)	0.30	-10.6	0.32	426 (337, 538)	475 (388, 582)	396 (314, 501)	0.59	-7.0	0.55
**2-Hydroxyestradiol**	102 (82.6, 127)	111 (91.8, 134)	92.4 (74.5, 115)	0.27	-9.4	0.33	100 (80.6, 124)	110 (90.4, 133)	94.3 (75.6, 118)	0.58	-5.7	0.60
**2-Methoxyestrone**	272 (222, 332)	271 (225, 326)	219 (183, 262)	**0.03**	-19.5	**0.04**	257 (211, 314)	264 (219, 318)	229 (191, 276)	0.25	-10.9	0.30
Conjugated	162 (129, 204)	166 (133, 208)	144 (118, 177)	0.20	-11.1	0.27	153 (121, 194)	162 (130, 203)	151 (123, 185)	0.79	-1.3	0.90
Unconjugated	83.8 (64.0, 110)	78.5 (60.9, 101)	54.0 (40.7, 71.7)	**0.003**	-35.6	**0.003**	77.1 (59.1, 101)	75.6 (58.6, 97.6)	57.8 (43.3, 77.2)	0.06	-25.0	0.06
**2-Methoxyestradiol**	91.3 (70.1, 119)	83.5 (64.6, 108)	76.8 (61.0, 96.7)	0.05	-15.9	0.09	86.9 (66.9, 113)	81.5 (63.1, 105)	80.3 (63.5, 102)	0.30	-7.6	0.49
Conjugated	76.5 (57.2, 102)	73.0 (55.1, 96.7)	64.7 (50.3, 83.3)	0.06	-15.4	0.15	70.6 (53.1, 94.0)	70.5 (53.4, 93.1)	69.0 (53.3, 89.3)	0.51	-2.3	0.85
Unconjugated	9.37 (7.71, 11.4)	8.59 (7.20, 10.2)	8.17 (6.79, 9.83)	0.29	-12.8	0.19	9.13 (7.50, 11.1)	8.49 (7.08, 10.2)	8.34 (6.92, 10.1)	0.57	-8.7	0.40
**2-Hydroxyestrone-3-methyl ether**	44.1 (35.9, 54.1)	42.8 (35.0, 52.3)	35.4 (29.1, 43.0)	**0.03**	-19.7	**0.03**	42.7 (34.7, 52.5)	42.1 (34.4, 51.6)	36.5 (29.9, 44.4)	0.13	-14.5	0.13
**4-Hydroxyestrone**	58.3 (46.0, 73.8)	64.3 (52.5, 78.8)	51.9 (41.1, 65.5)	0.31	-11.0	0.30	57.4 (45.2, 72.9)	63.8 (51.9, 78.4)	52.6 (41.5, 66.8)	0.56	-8.4	0.48
**4-Methoxyestrone**	28.6 (23.2, 35.1)	28.3 (23.5, 34.0)	22.8 (19.1, 27.3)	**0.04**	-20.3	**0.03**	27.5 (22.2, 33.9)	27.7 (23.0, 33.5)	23.6 (19.7, 28.4)	0.24	-14.2	0.17
**4-Methoxyestradiol**	12.1 (9.06, 16.2)	10.9 (8.29, 14.3)	10.8 (8.34, 14.0)	0.19	-10.7	0.31	11.5 (8.59, 15.5)	10.6 (8.09, 13.9)	11.3 (8.70, 14.7)	0.62	-1.7	0.89
**16α-Hydroxyestrone**	228 (179, 291)	257 (209, 316)	208 (164, 264)	0.42	-8.8	0.42	224 (175, 287)	255 (206, 314)	211 (165, 270)	0.72	-5.8	0.63
**Estriol**	1093 (832, 1438)	1172 (928, 1479)	985 (761, 1274)	0.47	-9.9	0.39	1077 (814, 1423)	1163 (920, 1470)	999 (769, 1298)	0.74	-7.2	0.57
Conjugated	937 (703, 1248)	1031 (806, 1319)	846 (646, 1107)	0.49	-9.7	0.43	917 (683, 1230)	1021 (798, 1308)	861 (655, 1130)	0.78	-6.1	0.65
Unconjugated	127 (101, 160)	128 (104, 156)	114 (90.6, 144)	0.53	-10.2	0.31	123 (96.8, 155)	126 (103, 154)	117 (92.9, 148)	0.94	-4.9	0.70
**16-Ketoestradiol**	265 (205, 341)	290 (235, 359)	245 (193, 312)	0.54	-7.5	0.53	259 (200, 336)	288 (232, 356)	250 (195, 320)	0.85	-3.5	0.78
**16-Epiestriol**	80.8 (64.3, 102)	89.4 (73.6, 109)	73.2 (59.0, 90.9)	0.44	-9.4	0.38	79.0 (62.6, 99.8)	88.4 (72.6, 108)	74.7 (59.7, 93.5)	0.80	-5.4	0.65
**17-Epiestriol**	54.9 (43.3, 69.6)	56.0 (45.3, 69.2)	51.3 (41.1, 64.0)	0.55	-6.6	0.55	53.6 (41.8, 68.6)	55.4 (44.6, 68.8)	52.4 (41.5, 66.1)	0.90	-2.2	0.87

[a] Model 1: Adjusted for age at blood draw (<55, 55-59, 60-64, 65-69, 70-74, 75-79 years), blood draw year (1993-1996, 1997-1998), race (white, non-white), smoking status (never, former, current), time since menopause (<10, 10-19, ≥20 years, missing), moderate- to vigorous-intensity physical activity (0, 0.1-9.9, ≥10 MET-hr/wk).

[b] Model 1 + current BMI (kg/m^2, continuous).

[c] p-trend was estimated using the Wald test for continuous waist-to-hip ratio.

[d] %Δ indicates the percent change in estrogens/estrogen metabolite levels comparing women with waist-to-hip ratio ≥0.83 vs. <0.76 and was estimated by taking the ratio of the geometric mean difference in estrogens/estrogen metabolite levels between women with waist-to-hip ratio ≥0.83 vs. <0.76 to the geometric mean of women with waist-to-hip ratio <0.76, multiplied by 100.

[e] p-diff was estimated using the Wald test and indicates a p-value for comparing estrogens/estrogen metabolite levels of women with waist-to-hip ratio ≥0.83 vs. <0.76.

[f] Weighted N reflects weighted counts and refer to the study cohort.

Note: All False Discovery Rate q-values>0.05.

Abbreviations: CI=confidence interval.
