# Supplementary material for: Anthropometric measures and serum estrogen metabolism in postmenopausal women: the Women’s Health Initiative Observational Study
Source: Breast Cancer Res. 2017 Mar 11;19:28. doi: 10.1186/s13058-017-0810-0 (PMC5346241; doi:10.1186/s13058-017-0810-0)
Supplement: Additional file 5: Table S3. — Geometric means (pmol/L) and 95% CIs of serum estrogens/estrogen metabolites by BMI at age 18 years in postmenopausal women in the Women’s Health Initiative Observational Study. (PDF 94 kb) [file 13058_2017_810_MOESM5_ESM.pdf]

Table S3. Geometric means (pmol/L) and 95% confidence intervals (CI) of serum estrogens/estrogen metabolites by BMI at age 18 in postmenopausal women: the Women's Health Initiative Observational Study

	Never and former menopausal hormone therapy users						Current menopausal hormone therapy users					
	Geometric means (95% CI)[a]						Geometric means (95% CI)[a]					
	<20 kg/m^2	20-21.9 kg/m^2	≥22 kg/m^2	p-trend[b]	%Δ[c]	p-diff[d]	<20 kg/m^2	20-21.9 kg/m^2	≥22 kg/m^2	p-trend[b]	%Δ[c]	p-diff[d]
**Median (kg/m^2)**	18.7	20.9	23.9				18.8	20.8	23.4			
**N**	412	300	221				371	334	154			
**Weighted N[e]**	14325	9690	6133				10882	9510	4269			
**Estrone**	269 (223, 324)	300 (246, 364)	322 (260, 399)	0.27	19.7	0.09	2927 (2208, 3880)	3057 (2350, 3978)	3384 (2388, 4794)	0.42	15.6	0.36
Conjugated	206 (167, 254)	235 (189, 291)	248 (195, 314)	0.29	20.4	0.11	2649 (1981, 3542)	2844 (2171, 3726)	3097 (2151, 4460)	0.43	16.9	0.35
Unconjugated	55.7 (48.6, 63.9)	55.3 (47.8, 64.0)	65.1 (54.6, 77.6)	0.23	16.9	0.08	205 (167, 252)	211 (172, 259)	231 (176, 302)	0.54	12.7	0.32
**Estradiol**	49.8 (40.4, 61.5)	50.7 (40.8, 63.1)	64.1 (49.3, 83.4)	**0.04**	28.7	**0.03**	391 (296, 515)	444 (336, 588)	441 (311, 625)	0.50	12.8	0.42
Conjugated	32.6 (26.1, 40.8)	33.2 (26.7, 41.3)	42.3 (32.4, 55.1)	**0.04**	29.8	**0.03**	322 (237, 436)	370 (273, 501)	377 (257, 555)	0.47	17.1	0.34
Unconjugated	12.7 (10.0, 16.2)	13.3 (10.3, 17.2)	16.2 (12.0, 21.7)	0.08	27.6	0.08	40.1 (32.0, 50.3)	46.9 (37.0, 59.5)	43.6 (32.9, 57.9)	0.55	8.7	0.50
**2-Hydroxyestrone**	59.5 (50.3, 70.5)	67.5 (57.5, 79.2)	66.9 (56.2, 79.6)	0.32	12.4	0.16	434 (352, 534)	412 (335, 507)	449 (346, 583)	0.54	3.5	0.77
**2-Hydroxyestradiol**	15.2 (12.9, 17.9)	16.9 (14.3, 19.9)	16.4 (13.9, 19.4)	0.55	7.9	0.32	99.8 (82.2, 121)	98.9 (81.8, 120)	105 (82.0, 134)	0.39	5.2	0.65
**2-Methoxyestrone**	39.9 (34.9, 45.6)	39.2 (34.3, 44.8)	42.5 (36.4, 49.8)	0.42	6.5	0.43	251 (214, 296)	251 (211, 297)	253 (198, 324)	0.90	0.8	0.95
Conjugated	28.9 (24.9, 33.4)	28.6 (24.7, 33.1)	31.0 (26.1, 36.8)	0.34	7.3	0.43	158 (130, 192)	159 (129, 197)	152 (115, 201)	0.89	-3.8	0.75
Unconjugated	10.1 (8.76, 11.7)	9.83 (8.47, 11.4)	10.5 (8.89, 12.5)	0.90	4.0	0.69	68.6 (53.4, 88.1)	70.0 (54.2, 90.5)	72.2 (51.0, 102)	0.95	5.2	0.75
**2-Methoxyestradiol**	12.0 (10.0, 14.4)	13.1 (10.8, 15.9)	13.0 (10.6, 15.8)	0.39	8.3	0.42	83.4 (66.2, 105)	83.7 (65.6, 107)	87.1 (66.5, 114)	0.16	4.4	0.69
Conjugated	9.55 (7.82, 11.7)	10.4 (8.44, 12.9)	10.2 (8.25, 12.7)	0.47	6.8	0.49	71.2 (55.3, 91.8)	72.2 (55.3, 94.2)	73.0 (54.4, 97.9)	0.25	2.5	0.84
Unconjugated	1.95 (1.66, 2.28)	2.11 (1.76, 2.54)	2.19 (1.81, 2.65)	0.32	12.3	0.21	8.40 (7.16, 9.85)	8.91 (7.47, 10.6)	8.61 (6.77, 11.0)	0.55	2.5	0.83
**2-Hydroxyestrone-3-methyl ether**	7.05 (6.11, 8.13)	7.50 (6.55, 8.59)	7.72 (6.56, 9.09)	0.51	9.5	0.26	40.2 (33.6, 48.1)	38.3 (31.5, 46.5)	38.5 (30.3, 49.0)	0.67	-4.2	0.69
**4-Hydroxyestrone**	7.31 (6.16, 8.68)	8.30 (7.03, 9.80)	8.24 (6.89, 9.84)	0.36	12.7	0.17	57.8 (46.7, 71.4)	54.7 (44.5, 67.3)	60.7 (46.5, 79.1)	0.46	5.0	0.69
**4-Methoxyestrone**	4.11 (3.56, 4.73)	4.18 (3.64, 4.79)	4.34 (3.70, 5.10)	0.56	5.6	0.50	25.8 (21.7, 30.7)	25.8 (21.7, 30.6)	27.1 (21.7, 34.0)	0.62	5.0	0.65
**4-Methoxyestradiol**	1.76 (1.48, 2.11)	1.83 (1.54, 2.18)	1.78 (1.47, 2.17)	0.75	1.1	0.92	11.4 (8.84, 14.8)	11.1 (8.56, 14.5)	11.2 (8.29, 15.1)	0.41	-1.8	0.87
**16α-Hydroxyestrone**	29.7 (24.8, 35.5)	33.9 (28.7, 40.1)	32.9 (27.4, 39.6)	0.44	10.8	0.23	229 (183, 285)	220 (178, 273)	245 (187, 321)	0.43	7.0	0.56
**Estriol**	125 (105, 149)	142 (120, 168)	140 (116, 170)	0.32	12.0	0.22	1062 (835, 1352)	1063 (843, 1340)	1128 (841, 1513)	0.48	6.2	0.66
Conjugated	96.7 (79.8, 117)	113 (94.1, 136)	109 (88.2, 134)	0.27	12.7	0.24	918 (713, 1181)	923 (724, 1177)	978 (715, 1337)	0.58	6.5	0.66
Unconjugated	25.1 (21.6, 29.3)	26.7 (23.0, 31.0)	28.3 (23.7, 33.7)	0.48	12.7	0.19	118 (96.9, 145)	123 (100, 152)	122 (94.3, 157)	0.86	3.4	0.81
**16-Ketoestradiol**	31.6 (26.4, 37.9)	36.1 (30.5, 42.8)	35.0 (28.9, 42.4)	0.51	10.8	0.27	263 (211, 328)	256 (206, 318)	291 (221, 384)	0.37	10.6	0.43
**16-Epiestriol**	14.0 (12.0, 16.5)	15.4 (13.2, 18.1)	14.9 (12.6, 17.6)	0.41	6.4	0.45	78.6 (64.6, 95.7)	80.9 (66.1, 98.9)	80.0 (62.3, 103)	0.67	1.8	0.88
**17-Epiestriol**	11.6 (9.97, 13.5)	13.1 (11.1, 15.4)	12.6 (10.7, 14.8)	0.46	8.6	0.32	52.7 (42.9, 64.9)	52.8 (42.7, 65.2)	59.0 (46.1, 75.6)	0.18	12.0	0.32

[a] Adjusted for age at blood draw (<55, 55-59, 60-64, 65-69, 70-74, 75-79 years), blood draw year (1993-1996, 1997-1998), race (white, non-white), smoking status (never, former, current), time since menopause (<10, 10-19, ≥20 years, missing).

[b] p-trend was estimated using the Wald test for continuous BMI at age 18 (kg/m^2).

[c] %Δ indicates the percent change in estrogens/estrogen metabolite levels comparing women with BMI at age 18 years ≥22 vs. <20 kg/m^2 and was estimated by taking the ratio of the geometric mean difference in estrogens/estrogen metabolite levels between women with BMI at age 18 years ≥22 vs. <20 kg/m^2 to the geometric mean of women with BMI at age 18 years <20 kg/m^2, multiplied by 100.

[d] p-diff was estimated using the Wald test and indicates a p-value for comparing estrogens/estrogen metabolite levels of women with BMI at age 18 years ≥22 vs. <20 kg/m^2.

[e] Weighted N reflects weighted counts and refer to the study cohort.

Note: All False Discovery Rate q-values>0.05.

Abbreviations: BMI=body mass index, CI=confidence interval.
