# Supplementary material for: Anthropometric measures and serum estrogen metabolism in postmenopausal women: the Women’s Health Initiative Observational Study
Source: Breast Cancer Res. 2017 Mar 11;19:28. doi: 10.1186/s13058-017-0810-0 (PMC5346241; doi:10.1186/s13058-017-0810-0)
Supplement: Additional file 6: Table S4. — Geometric means (pmol/L) and 95% CIs of serum estrogens/estrogen metabolites by current BMI in postmenopausal women not using menopausal hormone therapy among control subjects only in the Women’s Health Initiative Observational Study. (PDF 103 kb) [file 13058_2017_810_MOESM6_ESM.pdf]

Table S4. Geometric means (pmol/L) and 95% confidence intervals (CI) of serum estrogens/estrogen metabolites by current BMI in postmenopausal women not using menopausal hormone therapy among controls only: the Women's Health Initiative Observational Study

	**Model 1[a]**						**Model 1 + unconjugated estradiol[b]**					
	**Geometric means (95% CI)**						**Geometric means (95% CI)[c]**					
	**<25 kg/m²**	**25-29.9 kg/m²**	**≥30 kg/m²**	**p-trend[c]**	**%Δ[d]**	**p-diff[e]**	**<25 kg/m²**	**25-29.9 kg/m²**	**≥30 kg/m²**	**p-trend[c]**	**%Δ[d]**	**p-diff[e]**
**Median (kg/m²)**	22.5	27.1	33.4				22.5	27.1	33.4			
**N**	193	143	113				193	143	113			
**Weighted N[f]**	13419	9200	7692				13419	9200	7692			
**Estrone**	238 (201, 282)	335 (282, 398)	429 (353, 521)	**<0.001****	80.3	**<0.001****	277 (236, 325)	304 (262, 354)	289 (246, 338)	0.90	4.3	0.65
Conjugated	178 (147, 216)	264 (218, 319)	345 (279, 428)	**<0.001****	93.8	**<0.001****	208 (174, 250)	238 (201, 283)	229 (191, 275)	0.58	10.1	0.36
Unconjugated	51.1 (44.7, 58.5)	62.1 (55.0, 70.3)	75.2 (64.5, 87.8)	**<0.001****	47.2	**<0.001****	58.1 (51.9, 65.0)	57.3 (52.6, 62.4)	54.0 (48.9, 59.6)	0.14	-7.1	0.26
**Estradiol**	45.7 (37.5, 55.8)	58.8 (48.5, 71.4)	73.5 (59.4, 90.9)	**<0.001****	60.8	**<0.001****	53.5 (46.2, 61.9)	53.2 (46.3, 61.2)	48.7 (42.8, 55.5)	0.37	-9.0	0.21
Conjugated	30.8 (25.0, 38.1)	40.0 (32.3, 49.6)	45.6 (36.1, 57.7)	**<0.001****	48.1	**<0.001****	34.8 (28.6, 42.5)	37.0 (30.8, 44.5)	33.2 (27.6, 39.9)	0.95	-4.6	0.63
Unconjugated	10.1 (8.11, 12.7)	14.8 (12.3, 17.9)	23.4 (18.7, 29.3)	**<0.001****	131.	**<0.001****	NA	NA	NA	NA	NA	NA
**2-Hydroxyestrone**	55.4 (47.5, 64.6)	71.4 (61.2, 83.2)	82.7 (70.1, 97.6)	**<0.001****	49.3	**<0.001****	61.0 (52.5, 71.0)	67.1 (58.3, 77.2)	64.2 (54.8, 75.2)	0.87	5.2	0.57
**2-Hydroxyestrdaiol**	14.2 (12.2, 16.5)	17.7 (15.1, 20.7)	20.3 (17.2, 24.1)	**<0.001****	43.0	**<0.001****	15.5 (13.4, 18.0)	16.7 (14.4, 19.3)	16.0 (13.6, 18.8)	0.72	3.2	0.73
**2-Methoxyestrone**	37.7 (33.0, 42.9)	43.4 (38.2, 49.4)	47.5 (41.3, 54.6)	**0.001****	26.0	**0.002****	41.3 (36.7, 46.5)	40.9 (36.7, 45.6)	37.3 (33.0, 42.1)	0.05	-9.7	0.16
Conjugated	27.4 (23.7, 31.6)	32.0 (27.7, 37.0)	34.4 (29.4, 40.2)	**0.003****	25.5	**0.006****	29.7 (26.1, 33.8)	30.4 (26.8, 34.4)	27.8 (24.1, 32.0)	0.26	-6.4	0.43
Unconjugated	9.66 (8.37, 11.1)	10.4 (9.07, 12.0)	11.6 (9.99, 13.5)	**0.01***	20.1	**0.03***	10.9 (9.53, 12.5)	9.66 (8.46, 11.0)	8.44 (7.34, 9.70)	**<0.001****	-22.6	**0.001***
**2-Methoxyestradiol**	11.4 (9.58, 13.5)	13.4 (11.5, 15.7)	16.4 (13.6, 19.8)	**<0.001****	43.9	**<0.001****	12.6 (10.9, 14.7)	12.6 (11.0, 14.4)	12.5 (10.5, 14.9)	0.53	-0.8	0.92
Conjugated	8.99 (7.43, 10.9)	10.7 (8.82, 12.9)	13.4 (10.9, 16.5)	**<0.001****	49.1	**<0.001****	9.93 (8.32, 11.9)	10.0 (8.42, 11.9)	10.3 (8.47, 12.6)	0.87	3.7	0.69
Unconjugated	1.90 (1.63, 2.21)	2.20 (1.91, 2.54)	2.35 (2.01, 2.73)	**<0.001****	23.7	**0.006****	2.15 (1.92, 2.40)	2.03 (1.80, 2.30)	1.70 (1.53, 1.90)	**0.001***	-20.9	**<0.001****
**2-Hydroxyestrone-3-methyl ether**	6.72 (5.89, 7.67)	7.91 (6.86, 9.12)	8.70 (7.51, 10.1)	**0.006****	29.5	**0.001****	7.23 (6.31, 8.29)	7.55 (6.60, 8.65)	7.19 (6.16, 8.40)	0.28	-0.6	0.95
**4-Hydroxyestrone**	6.74 (5.77, 7.87)	8.65 (7.43, 10.1)	10.2 (8.52, 12.1)	**<0.001****	51.3	**<0.001****	7.39 (6.34, 8.61)	8.16 (7.08, 9.40)	7.98 (6.71, 9.48)	0.92	8.0	0.42
**4-Methoxyestrone**	3.91 (3.42, 4.47)	4.34 (3.86, 4.89)	4.85 (4.14, 5.67)	**0.02***	24.0	**0.01***	4.21 (3.67, 4.82)	4.15 (3.71, 4.64)	4.01 (3.42, 4.70)	0.22	-4.8	0.61
**4-Methoxyestradiol**	1.65 (1.40, 1.95)	1.86 (1.61, 2.15)	2.21 (1.84, 2.66)	**<0.001****	33.9	**0.001****	1.81 (1.55, 2.12)	1.75 (1.53, 2.00)	1.73 (1.45, 2.08)	0.31	-4.4	0.65
**16α-Hydroxyestrone**	27.4 (23.2, 32.3)	35.7 (30.4, 41.9)	41.5 (34.8, 49.6)	**<0.001****	51.5	**<0.001****	30.2 (25.7, 35.5)	33.5 (28.9, 38.8)	32.1 (27.0, 38.1)	0.96	6.3	0.52
**Estriol**	113 (96.7, 133)	155 (133, 180)	178 (150, 211)	**<0.001****	57.5	**<0.001****	126 (108, 146)	145 (126, 165)	135 (115, 160)	0.90	7.1	0.44
Conjugated estriol	86.6 (72.8, 103)	124 (105, 146)	142 (118, 172)	**<0.001****	64.0	**<0.001****	96.2 (81.3, 114)	116 (99.1, 135)	108 (90.0, 130)	0.57	12.3	0.27
Unconjugated	23.1 (20.0, 26.6)	27.3 (23.9, 31.3)	33.0 (28.2, 38.6)	**<0.001****	42.9	**<0.001****	25.7 (22.6, 29.1)	25.6 (22.7, 28.8)	25.1 (21.7, 29.0)	0.33	-2.3	0.78
**16-Ketoestradiol**	29.0 (24.5, 34.2)	38.1 (32.3, 44.9)	44.3 (36.9, 53.0)	**<0.001****	52.8	**<0.001****	32.0 (27.2, 37.6)	35.7 (30.7, 41.6)	34.2 (28.7, 40.7)	0.99	6.9	0.48
**16-Epiestriol**	12.8 (11.1, 14.9)	16.3 (13.9, 19.2)	19.2 (16.4, 22.6)	**<0.001****	50.0	**<0.001****	14.0 (12.1, 16.2)	15.4 (13.3, 17.9)	15.3 (13.1, 17.8)	0.74	9.3	0.28
**17-Epiestriol**	10.7 (9.32, 12.3)	13.4 (11.6, 15.6)	16.4 (14.0, 19.3)	**<0.001****	53.3	**<0.001****	11.7 (10.3, 13.4)	12.7 (11.1, 14.5)	13.0 (11.1, 15.2)	0.69	11.1	0.19

[a] Model 1: Adjusted for age at blood draw (<55, 55-59, 60-64, 66-69, 70-74, 75-79 years), blood draw year (1993-1996, 1997-1998), race (white, non-white), smoking status (never, former, current), time since menopause (<10, 10-19, ≥20 years, missing), physical activity (0, 0.1-9.9, ≥10 MET-hr/wk).

[b] Model 1 + unconjugated estradiol (log-transformed, continuous).

[c] p-trend was estimated using the Wald test for continuous BMI (kg/m²).

[d] %Δ indicates the percent change in estrogens/estrogen metabolite levels comparing women with current BMI ≥30 vs. <25 kg/m² and was estimated by taking the ratio of the geometric mean difference in estrogens/estrogen metabolite levels between women with current BMI ≥30 vs. <25 kg/m² to the geometric mean of women with current BMI <25 kg/m², multiplied by 100.

[e] p-diff was estimated using the Wald test and indicates a p-value for comparing estrogens/estrogen metabolite levels of women with current BMI ≥30 vs. <25 kg/m².

[f] Weighted N reflects weighted counts and refer to the study cohort.

* indicates False Discovery Rate (FDR) q-value<0.05 and ≥0.01. ** indicates FDR q-value<0.01.

Abbreviations: BMI=body mass index, CI=confidence interval, NA=not applicable.
